# FLT3 inhibitors in acute myeloid leukaemia: assessment of clinical effectiveness, adverse events and future research—a systematic review and meta-analysis

**DOI:** 10.1186/s13643-020-01540-1

**Published:** 2020-12-07

**Authors:** S. Majothi, D. Adams, J. Loke, S. P. Stevens, K. Wheatley, J. S. Wilson

**Affiliations:** 1 Cancer Research Clinical Trials Unit, University of Birmingham, Birmingham, UK; 2 Kingsmead Scientific Services Ltd, High Wycombe, Buckinghamshire UK; 3 Department of Haematology, Queen Elizabeth Hospital, Birmingham, UK; 4 Institute of Cancer & Genomic Sciences, Robert Aitken Institute of Clinical Research, University of Birmingham, Birmingham, UK

**Keywords:** FLT3 inhibitors, AML, Systematic review, Meta-analysis, Survival, Adverse events, Sorafenib, Midostaurin, Gilteritinib, Quizartinib

## Abstract

**Background:**

FMS-like tyrosine kinase 3 (FLT3) is the most frequent mutation in AML. With two FLT3 inhibitors recently approved by the FDA (midostaurin and gilteritinib), there is a need to evaluate these targeted agents.

**Purpose:**

To assess the clinical effectiveness of FLT3 inhibitors in AML patients.

**Methods:**

Standard systematic review methods were utilised. Searches were conducted to July 2020 for completed and in-progress randomised controlled trials of FLT3 inhibitors in AML. A fixed-effect meta-analysis was undertaken.

**Results:**

Eight completed trials involving 2656 patients and assessing five different FLT3 inhibitors (sorafenib, lestaurtinib, midostaurin, gilteritinib and quizartinib) were included. The pooled results were as follows (FLT3 inhibitor/control): overall survival hazard ratio (HR) = 0.83 (95% confidence interval [CI] 0.75 to 0.92, *p* = 0.0005), event-free survival HR = 0.85 (95% CI 0.77 to 0.94, *p* = 0.002), relapse-free survival HR = 0.76 (95% CI 0.64 to 0.90, *p* = 0.001), complete remission relative risk (RR) = 1.11 (95% CI 1.00 to 1.22. *p* = 0.05) and 60-day mortality RR = 1.04 (95% CI 0.77 to 1.40, *p* = 0.79).

Relative risk of grade 3 and above vascular, dermatological, respiratory and hepatobiliary adverse events were found to be statistically significantly higher in the FLT3 inhibitor group compared to control, but the actual numbers of events were relatively small. Nineteen ongoing trials are still in progress, only one of which specifically targets older patients with AML.

**Conclusions:**

There is evidence to support the use of FLT3 inhibitors in patients with AML, but more data is needed to verify the optimum use of the drugs regarding type of inhibitor, disease stage and patient characteristics, not only in relation to disease control, but adverse events and quality of life. There are a large number of ongoing trials; therefore, the results of this review are not a fait accompli; thus, is it recommended that the review be updated in a couple of years’ time. Given the challenges in extracting the complete data set required to assess clinical effectiveness, it is highly recommended that ongoing and future trials improve transparency and consistency of reporting of all trial outcomes, particularly disease control and adverse events, to enable a global clinical effectiveness assessment.

**Systematic review registration:**

PROSPERO CRD42017055581

**Supplementary Information:**

The online version contains supplementary material available at 10.1186/s13643-020-01540-1.

## Background

With an incidence of over 20,000 in the USA, acute myeloid leukaemia (AML) is the most common acute leukaemia in adults [1, 2]. Prognosis is poor, with 5-year overall survival (OS) of 40% in patients 60 years or younger and just 20% in patients aged over 60 years [3].

FMS-like tyrosine kinase 3 (FLT3) is the most frequent mutation in AML, found in one-third of patients with de novo AML. FLT3 internal tandem duplications (ITD) occur in almost a quarter of newly diagnosed cases of AML and approximately 7% of FLT3 mutations are tyrosine kinase domain (TKD) point mutations [4, 5]. Patients with FLT3 mutations, particularly FLT3-ITD, have a poor prognosis with an increased risk of relapse [6]. FLT3 inhibitors are tyrosine kinase inhibitors and are classified into first- and second-generation inhibitors based on their kinase specificity and potency. First-generation inhibitors include midostaurin and sorafenib. Second-generation inhibitors include quizartinib and gilteritinib, which are thought to be more potent and have less off-target effects. A number of different FLT3 inhibitors have been developed in the last twenty years and trialled in various treatment stages (remission induction, consolidation, maintenance, and in a relapsed/refractory setting) with varying evidence of efficacy, and there are also a number of FLT3 inhibitors undergoing assessment in ongoing trials. Two FLT3 inhibitors (midostaurin and gilteritinib) have recently been approved by the U.S. Food and Drug Administration (FDA) for use in patients with FLT3-mutated AML [7, 8]. Therefore, it is timely to undertake a systematic review and meta-analysis to assess the clinical effectiveness of FLT3 inhibitors in the treatment of patients with AML.

## Aim

To investigate the clinical effectiveness (including survival, disease response and adverse events) of FLT3 inhibitors in the treatment of patients with AML.

## Methods

Standard systematic review methods were employed and reported according to the Preferred Reporting Items for Systematic Reviews and Meta-Analyses (PRISMA) guidelines [9]. The review was based on an a priori protocol registered on PROSPERO (CRD42017055581) [10].

### Study inclusion criteria

The review included randomised controlled trials (RCTs) assessing the clinical effectiveness of any FLT3 inhibitor in patients of all ages with any type of AML. Comparators could include either standard care, another experimental comparator or placebo.

### Search strategy and study selection

A search of the bibliographic databases MEDLINE, EMBASE and MEDLINE In-Process (Ovid platform) was undertaken from 2000 to July 2020 using keywords such as ‘acute myeloid leukaemia’, ‘AML’, ‘fms-like tyrosine kinase 3’ and ‘FLT3’ (Supplementary File 1). The Ovid best balance filter of sensitivity and specificity was used, and no language restrictions were applied. Reference lists from included studies were citation-checked. Conference proceedings and ongoing trial databases were also searched between 2015 and July 2020 for completeness and to reduce the risk of publication and reporting bias. Study selection was undertaken by two reviewers independently with disagreements resolved by discussion.

### Data extraction and quality assessment

Data were extracted onto standardised and piloted forms. Relevant outcomes included OS, event-free survival (EFS), relapse-free survival (RFS), complete remission (CR) (including its variants complete remission with incomplete recovery [CRi], complete remission with incomplete platelet recovery [CRp] and overall response rate [ORR = CR + CRi/CRp]), early death, 30-day mortality, 60-day mortality, treatment-related mortality and adverse events (AEs). The Cochrane Risk of Bias Tool for RCTs was used for quality assessment [11]. Data extraction and quality assessment were based on information from published studies, conference abstracts, protocols and contact with study authors where relevant and undertaken by one reviewer (SM) with a second reviewer checking (DA/JW/SS).

Ongoing trials and conference abstracts were tabulated with trial characteristics, completion dates and results, where available.

### Statistical analyses

Time-to-event data were extracted based on methods from Tierney [12] and Parmar [13]: hazard ratios (HR), risk ratios (RR), 95% confidence intervals (CI), *p* values and survival proportions/events were used to calculate observed minus expected data and variance. Data were extracted from Kaplan-Meier curves where appropriate.

A fixed-effect meta-analysis was undertaken using Review Manager Version 5.4 [14]. Main analyses were based on uncensored data. Treatment effect was measured using HR or RR as appropriate; a *p* value of less than 0.05 indicated statistical significance. Heterogeneity of treatment effect was measured using the *χ*^2^ and *I*^2^ statistics and was explored using subgroup analysis, utilising the test for subgroup differences. Publication bias was tested for the outcome OS. Subgroup analyses investigated the effects of FLT3 inhibitor type, FLT3 mutation, disease stage, and age on OS, EFS and RFS, where data was available, with a sensitivity analysis undertaken for censored populations. Censoring was defined from the point when patients underwent stem cell transplantation (SCT). In trials lacking EFS data, EFS was calculated (RFS + [N-CR], where *N* represents the total number of patients).

Grade 3 and above AEs as defined by the National Cancer Institute Common Toxicity Criteria (CTC version 3.0) were grouped by biological system and tabulated. Where more than one trial reported an AE, these were pooled using fixed-effect meta-analysis.

## Results

### Search results

**Fig. 1:**
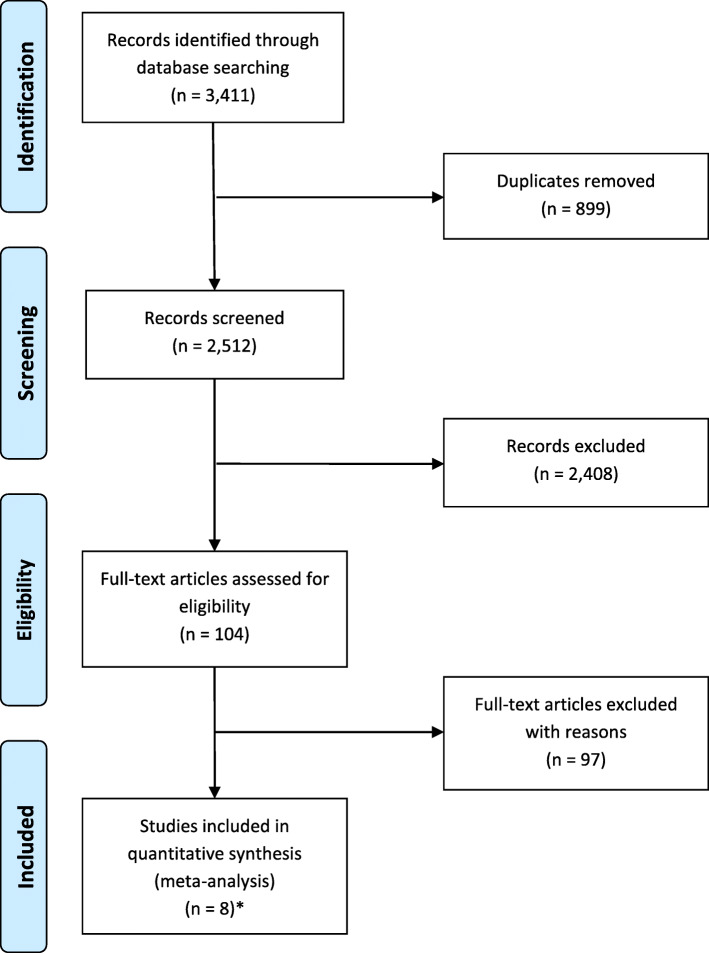
PRISMA flow diagram of study selection process. Asterisk indicates the eight completed randomised controlled trials reported in seven publications

The search identified 3411 references. From these, seven publications [15–21] reporting eight completed trials, and involving 2656 patients met the review inclusion criteria (Fig. 1).

Nineteen ongoing trials potentially involving 8429 patients also met the inclusion criteria (Supplementary Table 1). A search of conference proceedings found eight abstracts of six trials (Supplementary Table 2), most of which were trial protocols, but two included primary and final trial results (SORMAIN [22] and RADIUS [23]).

### Trial characteristics

All eight completed trials were multicentre; five were phase III [17, 19–21] and three phase II [15, 16, 18] trials. Five FLT3 inhibitors were investigated: sorafenib in two trials [15, 16], lestaurtinib in three [17, 18] and midostaurin [19], quizartinib [20] and gilteritinib [21] in one trial each. As part of their eligibility criteria, six trials required patients to have a FLT3 mutation [17–21].

Median follow-up was reported in seven trials and ranged from 17.8 to 59 months [15–17, 19–21]. Two trials included patients aged 18–60 years [15, 19] and one trial exclusively recruited patients aged over 60 years [16]. The remaining five trials included a small number of patients outside of their eligibility criteria, which was patients aged under 60 years for two trials [17] and aged 18 years or over for three trials [18, 20, 21].

An abridged table of characteristics can be found in Table 1, with full details in Supplementary Table 3. Of note, one paper reported two separate lestaurtinib trials [17]; in both, trial participants were recruited from two larger trials (AML15 and AML17) [24, 25], where patients were treated with a variety of chemotherapy treatments. Patients from AML15 [24] and AML17 [25] were eligible for lestaurtinib randomisation if they had a FLT3 mutation. Patients from AML15 received intensive chemotherapy with or without lestaurtinib and patients from AML17 received intensive chemotherapy with lestaurtinib or placebo [17]. Full chemotherapy and treatment schedules for all included trials are detailed in Supplementary Table 4.

**Table 1:** Characteristics of included studies

Author, year Trial name/number	Population selection criteria	Intervention *N* randomised and details	Control *N* randomised and details	Intervention baseline age (yrs) and type of AML	Control baseline age (yrs) and type of AML	Outcomes reported and median follow-up
Rollig C, 2015 [15] **SORAM**L; NCT00893373; Phase 2, Germany	Aged 18–60 yrs, newly diagnosed de novo or secondary AML (excluding APL)	Sorafenib 400 mg twice daily plus standard chemo—ind/cons/main up to 12 mths *N* = 138^a^	Placebo plus standard chemo *N* = 138^a^	Median age (range): 50 (43–46); de novo AML: NR; 2° AML: 10%; High-risk MDS: NR	Median age (range): 50 (44–55); de novo AML: NR; 2° AML: 15%; High-risk MDS: NR	1°: EFS 2°: RFS, OS, CR, tox FU: 36 mths
Serve H, 2013 [16] NCT00373373; Phase 2, Germany	Aged > 60 yrs, de novo or secondary AML or AML from MDS (excluding FAB type M3)	Sorafenib 400 mg twice daily plus intensive chemo—ind/cons *N* = 104^a^	Placebo plus intensive chemo *N* = 97^a^	Median age (range): 67.5 (61–78); de novo AML: 60%; 2° AML: 40%; High-risk MDS: NR	Median age (range): 69 (61–80); de novo AML: 61%; 2° AML: 39%; High-risk MDS: NR	1°: EFS 2°: OS, CR rate, tolerability FU: 29.3 mths
Knapper S, 2017 [17] **AML15**; ISRCTN17161961; Phase 3, UK Denmark, NZ	Aged < 60 yrs, de novo or secondary AML, FLT3 mutation (excluding APL)	Lestaurtinib 80 mg, twice daily after each of 4 courses of intensive chemo—ind/cons *N* = 88	Intensive chemo *N* = 87	Median age (range): 48 (16–66); de novo AML: 95%; 2° AML: 3%; High-risk MDS: 0%	Median age (range): 46 (16-65); de novo AML: 97%; 2° AML: 5%; High-risk MDS: 0%	1°: OS/RFS 2°: CR, CRi, OS, haem recovery times, tox, resource use FU: 50.5 mths
Knapper S, 2017 [17] **AML17**; ISRCTN55675535 Phase 3, UK Denmark, NZ	Aged < 60 yrs, de novo or secondary AML, FLT3 mutation (excluding APL)	Lestaurtinib 80 mg, twice daily plus 1st line intensive chemo—ind/cons *N* = 212	Placebo plus 1st line intensive chemo *N* = 113	Median age (range): 50 (5–68); de novo AML: 93%; 2° AML: 5%; High-risk MDS: 2%	Median age (range): 50 (6–65); de novo AML: 92%; 2° AML: 5%; High-risk MDS: 3%	1°: OS/RFS 2°: CR, CRi, OS, haem recovery times, tox, resource use FU: 50.5 mths
Levis M, 2011 [18] **Cephalon-204**; NCT00079482; Phase 2, Australia, Canada, EU, Israel, NZ, Russia, Ukraine, USA	Aged ≥ 18 yrs, AML with 1st relapse after 1st remission of 1–24 mths, FLT3 mutation	Salvage chemo followed by lestaurtinib 80 mg, twice daily *N* = 112	Salvage chemo *N* = 112	Median age (range): 59 (20–81); de novo AML: NR; 2° AML: NR; High-risk MDS: NR	Median age (range): 54 (21–79); de novo AML: NR; 2° AML: NR; High-risk MDS: NR	1°: CR, CRp 2°: OS, PR, tox, tolerability FU: not reported
Stone RM, 2017 [19] **RATIFY** calgb 10603; NCT00651261; Phase 3, Canada, USA	Aged 18–59 yrs, newly diagnosed AML, FLT3 mutation (excluding APL)	Midostaurin 50 mg twice daily plus standard chemo—ind/cons/main up to 12 mths *N* = 360	Placebo 50 mg twice daily plus standard chemo *N* = 357	Median age (range): 47.1 (19–60); de novo AML: NR; 2° AML: NR; High-risk MDS: NR	Median age (range): 48.6 (18–61); de novo AML: NR; 2° AML: NR; High-risk MDS: NR	1°: OS 2°: EFS, OS, CR rate, DFS, HCT rate FU: 59 mths
Perl AE, 2019 [21] **ADMIRAL;** NCT02421939; Phase 3, 14 countries	Aged > 18 yrs, relapsed or refractory AML, FLT3 mutation	Gilteritinib 120 mg, once daily in 28-day cycles *N* = 247	Salvage chemo *N* = 124	Median age (range): 62.0 (20.0–84.0); de novo AML: NR; 2° AML: NR; High-risk MDS: NR	Median age (range): 61.5 (19.0–85.0); de novo AML: NR; 2° AML: NR; High-risk MDS: NR	1°: OS, CR 2°: EFS, tox FU: 17.8 mths
Cortes JE, 2019 [20] **QuANTUM-R;** NCT02039726; Phase 3, 19 countries	Aged > 18 yrs, relapsed or refractory AML, FLT3-ITD mutation (excluding APL)	Quizartinib 20–60 mg as appropriate, once daily in continuous 28-day cycles *N* = 245	Salvage chemo *N* = 122	Median age (range): 55.0 (46.0–65.0); de novo AML: NR; 2° AML: NR; High-risk MDS: NR	Median age (range): 57.5 (44.0–66.0); de novo AML: NR; 2° AML: NR; High-risk MDS: NR	1°: OS 2°: EFS, CR, early death (30- and 60-day mortality) FU: 23.5 mths

*Abbreviations*: *AML* acute myeloid leukaemia, *APL* acute promyelocytic leukaemia, *chemo* chemotherapy, *cons* consolidation therapy, *CR* complete remission, *CRi* CR with incomplete haematologic recovery, *CRp* CR with incomplete platelet recovery, *DFS* disease-free survival, *EFS* event-free survival, *FAB* French-American British (classification system), *FLT3* fms-like tyrosine kinase 3, *FU* follow-up, *HCT* haematopoietic cell transplant, *ind* induction therapy, *main* maintenance therapy, *MDS* myelodysplastic syndrome, *mths* months, *NR* not reported, *NZ* New Zealand, *OS* overall survival, *PR* partial remission, *RFS* relapse-free survival, *tox* toxicity, *yrs* years, *1°* primary, *2°* secondary

^a^*N* includes patients who were randomised and untreated and/or not included in the individual trial analyses

### Outcomes

Definitions of outcomes were collated (Supplementary Table 5) and source of data was recorded (Supplementary Table 6).

#### Overall survival

**Fig. 2:**
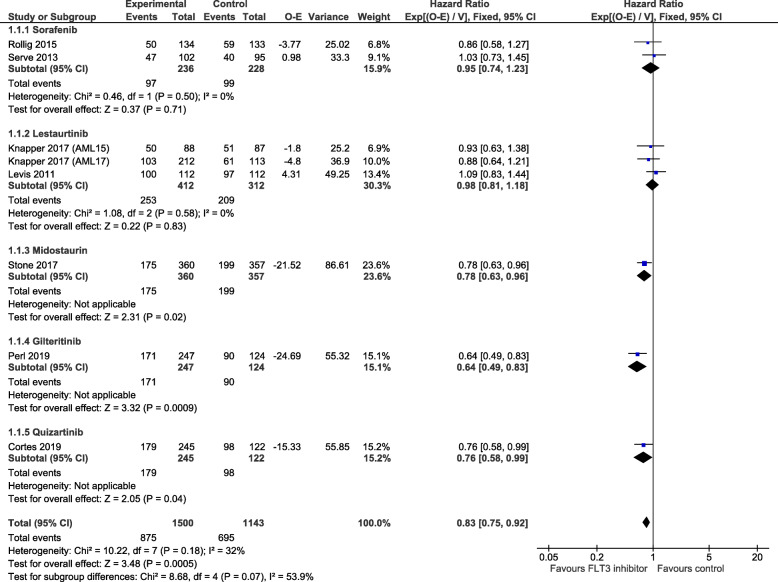
Forest plot of overall survival data (uncensored population), grouped by FLT3 inhibitor

The pooled HR, based on data from eight trials [15–21], favoured FLT3 inhibitors and was statistically significant (HR = 0.83, 95% CI 0.75 to 0.92, *p* = 0.0005, Fig. 2). There was moderate heterogeneity of treatment effect between trials (*I*^2^ = 32%). Use of midostaurin [19] showed a statistically significant increase in OS in newly diagnosed patients (HR = 0.78, 95% CI 0.63 to 0.96, *p* = 0.02), and both gilteritinib [21] and quizartinib [20] showed a statistically significant increase in OS in relapsed/refractory patients (HR = 0.64, 95% CI 0.49 to 0.83, *p* = 0.0009; and HR = 0.76, 95% CI 0.58 to 0.99, *p* = 0.04, respectively). No statistically significant improvement in OS was found with use of sorafenib or lestaurtinib. *χ*^2^ test for subgroup differences was not significant (*χ*^2^ = 8.68, *p* = 0.07, Fig. 2), but an *I*^2^ value of 53.9% suggests there was some subgroup differences between the FLT3 agents. A funnel plot of all eight studies did not indicate publication bias (Supplementary Figure 1).

#### Event-free survival

**Fig. 3:**
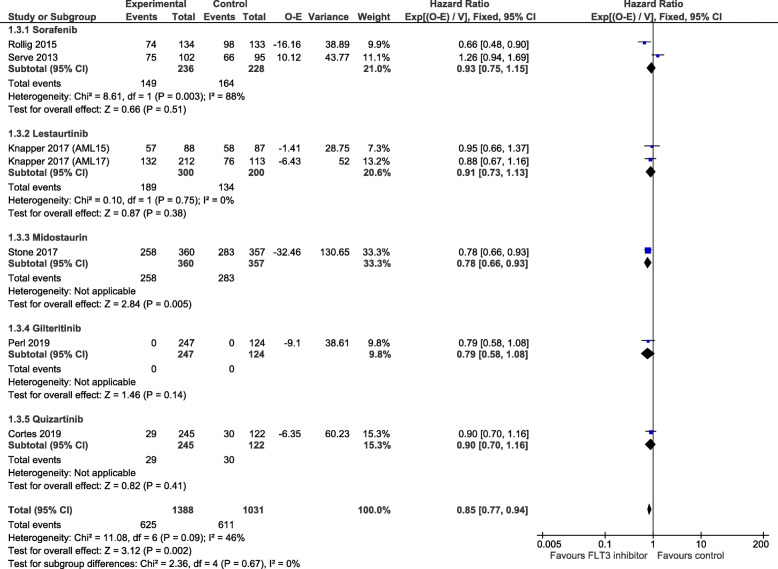
Forest plot of estimated event-free survival (calculated and reported data, uncensored population), grouped by FLT3 inhibitor

Response data and RFS data were used to estimate EFS in two lestaurtinib trials [17] that did not report EFS. One trial of lestaurtinib lacked both EFS and RFS data therefore estimation was not possible [18]. Pooling of data from seven trials [15–17, 19–21], including two for which EFS data was estimated [17], produced a statistically significant EFS in favour of FLT3 inhibitor group (HR = 0.85, 95% CI 0.77 to 0.94, *p* = 0.002, Fig. 3). EFS from five trials which directly reported EFS [15, 16, 19–21] was very similar (HR = 0.84, 95% CI 0.75 to 0.94, *p* = 0.002, Supplementary Figure 2). Overall heterogeneity was moderate for estimated EFS (seven trials: *I*^2^ = 46%) and substantial for reported EFS (five trials: *I*^2^ = 62%). Regarding individual FLT3 inhibitors, only midostaurin showed a benefit (HR = 0.78, 95% CI 0.66 to 0.93, *p* = 0.005, Fig. 3) [19], but the test for subgroup differences was not significant (*χ*^2^ = 2.36, *p* = 0.67, *I*^2^ = 0%).

#### Relapse-free survival

RFS was improved with the use of FLT3 inhibitors based on pooled data from four trials [15, 17, 19] (HR = 0.76, 95% CI 0.64 to 0.90, *p* = 0.001) with low heterogeneity (*I*^2^ = 16%, Supplementary Figure 3). Sorafenib and midostaurin showed statistically significant benefit in RFS. *χ*^2^ test for subgroup differences between the different FLT3 inhibitors was not statistically significant (*χ*^2^ = 3.50, *p* = 0.17), but *I*^2^ at 42.9% suggested a moderate difference of effect between different FLT3 agents.

#### Complete remission

Pooling data on CR from six trials [15, 16, 18–21] (of all FLT3 inhibitors) was borderline in favour of FLT3 inhibitors (RR = 1.11, 95% CI 1.00 to 1.22, *p* = 0.05, Supplementary Figure 4) with substantial heterogeneity (*I*^2^ = 63%). Six trials [16–18, 20, 21] (one sorafenib, three lestaurtinib, one gilteritinib and one quizartinib) were pooled using ORR data (i.e. CR + CRi or CR + CRp) with results statistically significantly in favour of FLT3 inhibitor (RR = 1.20, 95% CI 1.10 to 1.29, *p* < 0.00001, Supplementary Figure 5), although with considerable heterogeneity (*I*^2^ = 95%). Both analyses (CR and ORR) tested positive for subgroup differences.

### Subgroup analyses

#### Disease stage

Subgroup analyses based on disease stage resulted in a statistically significant effect in OS in favour of FLT3 inhibitor for patients with newly diagnosed or secondary disease [15–17, 19] and relapsed/refractory disease [18, 20, 21] (HR = 0.86, 95% CI 0.75 to 0.99, *p* = 0.03, *I*^2^ = 0%, 5 trials; and HR = 0.80, 95% CI 0.69 to 0.93, *p* = 0.005, *I*^2^ = 74%, 3 trials, respectively; Supplementary Figure 6). Test for subgroup differences was not significant (*χ*^2^ = 0.49, *p* = 0.49).

Estimated EFS was found to be statistically significant and in favour of FLT3 inhibitors for newly diagnosed or secondary disease [15–17, 19], but not in relapsed/refractory disease [20, 21] (HR = 0.85, 95% CI 0.76 to 0.96, *p* = 0.007, *I*^2^ = 63%, 5 trials; and HR = 0.86, 95% CI 0.70 to 1.04, *p* = 0.12, *I*^2^ = 0%, 2 trials, respectively; Supplementary Figure 7). Test for subgroup differences was not statistically significant (*χ*^2^ = 0.00, *p* = 0.99).

No outcome data for RFS was provided for the subgroup of relapsed patients.

#### Age

Three subgroups were categorised by age: 18–60 years, greater than 60 years and 18 years to unspecified. Patients aged 18–60 years [15, 17, 19] and older than 18 years [18, 20, 21] had statistically significantly improved OS (HR = 0.83, 95% CI 0.72 to 0.97, *p* = 0.02, *I*^2^ = 0%, 4 trials; and HR = 0.80, 95% CI 0.69 to 0.93, *p* = 0.005, *I*^2^ = 74%, 3 trials, respectively; Supplementary Figure 8), unlike patients older than 60 years [16] (HR = 1.03, 95% CI 0.73 to 1.45, *p* = 0.87, 1 trial). The test for subgroup differences was not statistically significant (*χ*^2^ = 1.75, *p* = 0.42, Supplementary Figure 8).

EFS in younger patients (18-60 years) also favoured FLT3 inhibitors (HR = 0.80, 95% CI 0.71 to 0.90, *p* = 0.0004, *I*^2^ = 0%, 4 trials, Supplementary Figure 9), but for 18 years to unspecified, which included older patients within the cohort [20, 21], this result was not statistically significant (HR = 0.86, 95% CI 0.70 to 1.04, *p* = 0.12, *I*^2^ = 0%, 2 trials). There was only one trial where only patients aged over 60 years were recruited [16], and the point estimate favoured control although this was not statistically significant (HR = 1.26, 95% CI 0.94 to 1.69, *p* = 0.13, Supplementary Figure 9). Heterogeneity was moderate (*I*^2^ = 46%) and test for subgroup differences was statistically significant (*χ*^2^ = 7.77, *p* = 0.02).

RFS data was not available for patients older than 60 years.

#### FLT3 mutation

Pooled analyses for patients with FLT3 mutations (i.e. FLT3-ITD, FLT3-TKD, etc.) were not possible given the limited data and inconsistencies in reporting, however individual trial results are shown in Supplementary Tables 7-15.

#### Censored population

Within included trials, patients were censored at the point of SCT. Six trials reported censored data for OS [15, 17–21] (HR = 0.82, 95% CI 0.73 to 0.93, *p* = 0.002, Supplementary Figure 10), which was in keeping with the results for the uncensored population; however, heterogeneity increased from an *I*^2^ of 32 to 44% and test for subgroup differences was borderline significant.

Only one sorafenib trial reported censored data for both EFS and RFS and again trends were in keeping with the pooled uncensored findings which were statistically significantly in favour of FLT3 inhibitor group (EFS, HR = 0.64, 95% CI 0.45 to 0.91, *p* = 0.01, and RFS, HR = 0.53, 95% CI 0.30 to 0.93, *p* = 0.03, data not shown) [15].

### Early mortality and adverse events

**Fig. 4:**
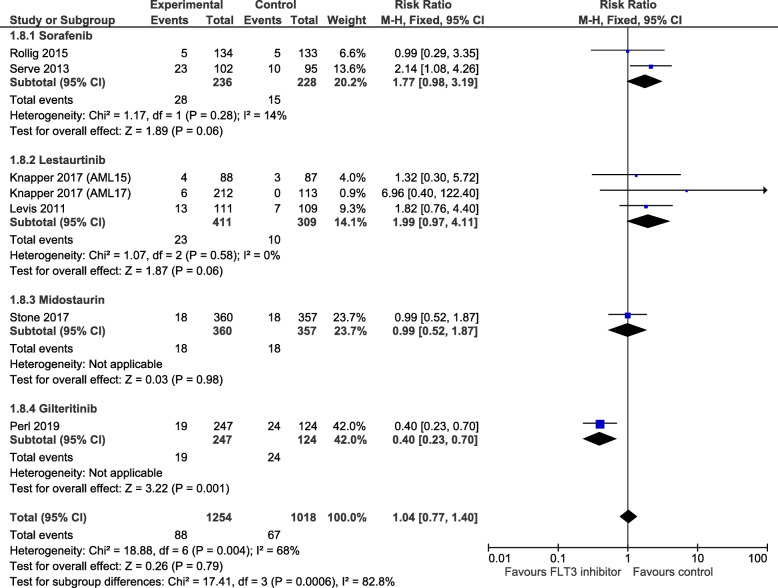
Forest plot of mortality data, grouped by FLT3 inhibitor. Pooled data includes 60-day mortality data, but one trial (Levis) reported 30-day mortality only, which was used in this analysis

Mortality data from each study was pooled (six trials reporting 60-day mortality [15–17, 19, 21] and one trial reporting 30-day mortality [18]). The actual number of deaths was low (88 in the FLT3 inhibitor group and 67 in the control group) and there was no statistically significant difference observed between FLT3 inhibitor group and control group (RR = 1.04, 95% CI 0.77 to 1.40, *p* = 0.79, Fig. 4). A moderate/substantial amount of heterogeneity was present (*I*^2^ = 68%) and test for subgroup differences was highly significant (*χ*^2^ = 17.41, *p* = 0.0006, Fig. 4). All mortality data is given in Supplementary Figure 11. Two trials [15, 16] reporting ‘early death’ (which was not defined and undistinguishable from 30- and 60-day mortality) showed a statistically significant increased risk in mortality with FLT3 inhibitor use compared to control (RR = 2.20, 95% CI 1.04 to 4.65, *p* = 0.04, *I*^2^ = 0%, Supplementary Figure 11). There was no evidence of effect for the use of FLT3 inhibitors in the reduction of 30-day or 60-day mortality (RR = 0.95, 95% CI 0.58 to 1.57, *p* = 0.85, *I*^2^ = 73%, 5 trials; and RR = 0.96, 95% CI 0.70 to 1.32, *p* = 0.80, *I*^2^ = 70%, 6 trials, respectively, Supplementary Figure 11). One trial reported treatment-related mortality (RR = 1.32, 95% CI 0.30 to 5.80, *p* = 0.71), although this was not statistically significant and numbers of events were low (4 events in sorafenib and 3 events in control group, data not shown) [15].

Grade 3 and above AEs are presented in Table 2 from seven trials [15–17, 19–21]. Reporting of AEs was extremely inconsistent between trials. Though percentages of some AEs were high, rates were similar between FLT3 inhibitor arms and their respective control groups. Pooling of AE data was undertaken according to biological system (Supplementary Figures 12-19). AEs from AML15 and AML17 were not reported separately in the primary lestaurtinib publication [17] and therefore have remained combined in the meta-analyses (shown as AML17 on data plots).

**Table 2:**
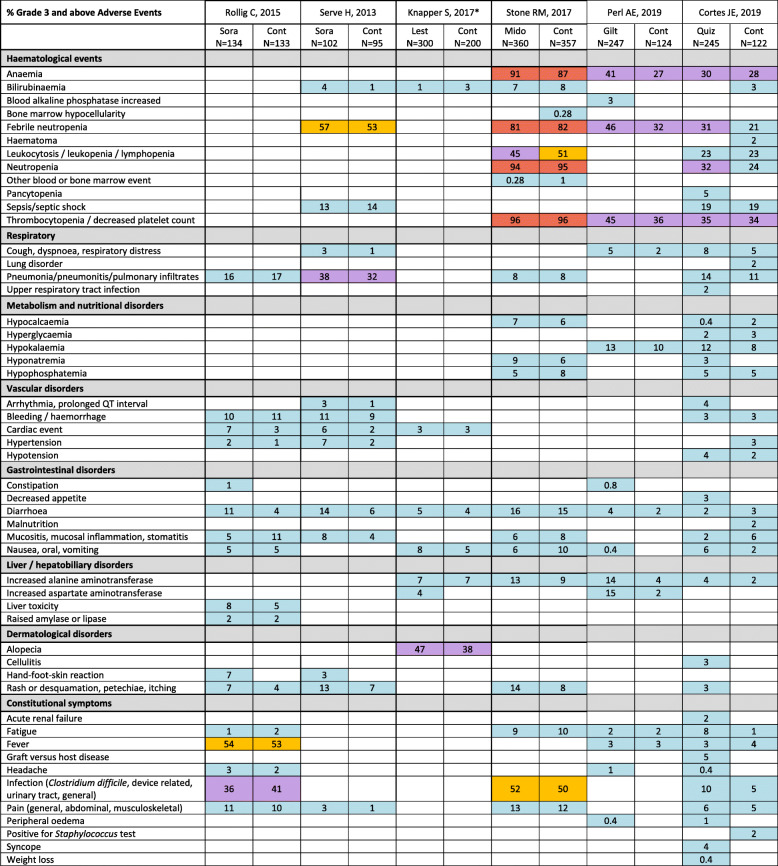
Percentage of grade 3 and above adverse events

Colour code: blue = 0 to 25%, purple = 26 to 50%, orange = 51 to 75% and red = 76 to 100%

*Abbreviations*: *cont* control, *gilt* gilteritinib, *lest* lestaurtinib, *mido* midostaurin, *quiz* quizartinib, *sora* sorafenib

*Includes patients from both AML15 and AML17 lestaurtinib trials following course 2 induction therapy

Statistically significant increases were seen in vascular AEs overall with use of FLT3 inhibitors (RR = 1.52, 95% CI 1.05 to 2.19, *p* = 0.02, *I*^2^ = 0%, Supplementary Figure 12) though individual vascular AEs (e.g. cardiac event, hypertension, etc.) were no different between FLT3 inhibitor and control groups. Dermatological AEs were also statistically significantly increased in FLT3 inhibitor group versus control group (RR = 1.55, 95% CI 1.28 to 1.87, *p* < 0.00001, *I*^2^ = 36%, Supplementary Figure 13) including all subgroups (alopecia *p* = 0.04, hand-foot-skin reaction *p* = 0.01, and rash, desquamation, petechiae, itching *p* = 0.0005). There were statistically significantly more respiratory AEs in FLT3 inhibitor group versus control group (RR = 1.25, 95% CI 1.03 to 1.52, *p* = 0.02, *I*^2^ = 0%, Supplementary Figure 14). Hepatobiliary AEs were also statistically significantly increased in the FLT3 inhibitor group compared to control group (RR = 1.98, 95% CI 1.46 to 2.69, *p* < 0.0001, *I*^2^ = 68%, Supplementary Figure 15). Overall, haematological AEs, metabolism and nutritional disorders, gastrointestinal disorders and constitutional symptoms were not significantly different between FLT3 inhibitor and control groups (RR = 1.03, 95% CI 1.00 to 1.07, *p* = 0.06, *I*^2^ = 51%, Supplementary Figure 16; RR = 1.10, 95% CI 0.86 to 1.41, *p* = 0.46, *I*^2^ = 17%, Supplementary Figure 17; RR = 1.07, 95% CI 0.88 to 1.29, *p* = 0.51, *I*^2^ = 35%, Supplementary Figure 18; RR = 1.05, 95% CI 0.95 to 1.17, *p* = 0.35, *I*^2^ = 0%, Supplementary Figure 19, respectively). Generally, the actual numbers of events were low across FLT3 inhibitor and control groups.

### Risk of bias

The overall quality of included trials was good to moderate, particularly with regards to random sequence generation and allocation concealment. Attrition bias was noted in both sorafenib trials [15, 16] whereby not all randomised patients were included in the intention-to-treat analyses; however, the numbers excluded were small. Outcome reporting bias was identified in two trials [15, 18] where outcomes stated in the protocol/methods were not reported and/or outcomes not predefined in protocol/methods were reported (Supplementary Figures 20 and 21).

### Ongoing trials

Nineteen ongoing RCTs were identified, with predicted study completion dates ranging from 2018 to 2028. Of these, three are investigating sorafenib, five midostaurin, five gilteritinib and one quizartinib. All of the sorafenib trials involve patients with newly diagnosed AML. Two (NCT01371981, NCT03164057) are multi-arm and in younger people aged below 29 and 21 years, with an estimated sample size of 1750 and 200, respectively. The third (ACTRN12611001112954) involves patients aged 15–65 years and aims to recruit 99 patients. Three midostaurin trials involve patients with newly diagnosed AML. All involve adults, with one (NCT03092674) aiming to recruit patients older than 60 years. Of the five gilteritinib trials, one is newly diagnosed AML patients aged under 22 years; the remaining are in adult populations, one each in newly diagnosed, at first complete response, post SCT and relapsed and refractory populations. The ongoing quizartinib trial is in adults with newly diagnosed disease. The remaining ongoing trials are investigating: crenolanib (3 trials), ponatinib (1 trial) and nintedanib (1 trial), with estimated sample sizes ranging from 9 to 510 participants (Supplementary Table 1).

### Conference abstracts

Eight conference abstracts not related to the trials included in the main part of this review were identified. Six were protocols for ongoing trials. Two conference abstracts gave results, both were small trials. The RADIUS [23] trial randomised 60 patients aged between 18 and 70 years to midostaurin or standard care post SCT. RFS was 89% versus 79%, respectively at 18 months. The SORMAIN [22] trial assessed sorafenib also as maintenance therapy following allogeneic SCT in 83 patients. Median RFS was not reached in the sorafenib group and was 30.9 months in the placebo group, with a 2-year RFS of 85.0% and 53.3%, respectively (HR = 0.39, *p* = 0.0139, Supplementary Table 2).

## Discussion

The aim of this systematic review was to assess the clinical effectiveness of FLT3 inhibitors, as a class of drugs, in patients with any type of AML. Eight completed RCTs and 19 ongoing trials were identified from the review searches. Three FLT3 inhibitors, sorafenib, lestaurtinib and midostaurin, were first-generation and two, gilteritinib and quizartinib, were second-generation inhibitors. In five trials [15–17, 19], patients had a mix of primary, de novo and secondary AML, with FLT3 inhibitors given as part of induction and/or consolidation treatment in three trials [16, 17] plus maintenance up to 12 months in two [15, 19]. Three trials included patients with relapsed/refractory disease [18, 20, 21]. There was a wide age range within the trials with four trials [15, 17, 19] aiming to recruit patients aged 18 to 60 years, one over 60 years [16] and three recruiting patients aged 18 years or older with no upper age limit specified [18, 20, 21]. All but the two sorafenib trials required patients to have a FLT3 mutation [17–21].

The pooled HR for OS for all eight trials in the uncensored population showed a 17% benefit in survival, which was statistically significant [15–21]. A moderate level of heterogeneity was observed, which was explored using subgroup analysis. For type of FLT3 inhibitor, subgroup analysis indicated a slight difference of effect between inhibitors, with *χ*^2^ at 8.68 (*p* = 0.07) and *I*^2^ at 53.9%. Whilst all of the FLT3 inhibitors showed benefit as demonstrated by their subgroup point estimates, HRs for sorafenib and lestaurtinib were not statistically significant [15–18]. Interestingly, when censored data was pooled, whilst OS remained statistically significant, the sorafenib subgroup pooled estimate favoured control and of those favouring FLT3 only, gilteritinib remained statistically significant. This could be due to a clinical effect with SCT enhancing the survival benefit of FLT3 inhibitors whether in synergy or as an additional therapy, or it could be due to a statistical anomaly due to loss of power in the censored analysis. Subgroup analyses comparing disease stage—newly diagnosed or secondary AML versus relapsed or refractory disease—found no difference between subgroups, with both showing a benefit in favour of FLT3. However, there was substantial heterogeneity within the relapsed and refractory group (*I*^2^ = 74%), with lestaurtinib showing no benefit. With four ongoing trials in patients with relapsed disease, more data may establish if there is a benefit in relapsed/refractory patients. For subgroup analyses investigating age, a favourable HR for FLT3 inhibitors was demonstrated for patients younger than 60 years (HR = 0.83, 95% CI 0.72 to 0.97) and also in the group with the very wide age range from 18 years to unspecified (HR = 0.80, 95% CI 0.69 to 0.93). In the trial which excluded patients under 60 years, a non-significant difference in favour of control was observed (HR = 1.03, 95% CI 0.73 to 1.45). The test for subgroup differences did not indicate a subgroup difference, but heterogeneity was substantial in the 18 years to unspecified group suggesting that age may have a role in the effectiveness of FLT3 inhibitors as this grouping included patients in their eighties. Just one ongoing trial (NCT03092674; suspended June 2020) is specifically aimed at patients over 60 years, so there is a need to define and consider age in future trials.

Ultimately, survival improvements are the goal of therapy for patients with AML, but disease control is also an important aspect of care. EFS, RFS and response data can help to gauge disease control and the risk of relapse, which for patients with FLT3-ITD mutations is substantial. Both EFS (HR = 0.85, 95% CI 0.77 to 0.94) and RFS (HR = 0.76, 95% CI 0.64 to 0.90) suggest an anti-leukaemic effect, and both measures of response (complete response and overall response) also showed a pooled estimate in favour of FLT3; however, both also report substantial heterogeneity. Unfortunately, all of these results may have been affected by reporting bias, as not all of the trials reported all of these outcome measures.

Other biases may also have affected the trial results and interacted with the clinical heterogeneity within and across trials. For example, in the older patients in the Serve trial, there was a significant difference in patients stopping therapy in the sorafenib arm compared to the control arm due to toxicity or refusal (*p* < 0.001) [16], with the consequence that patients randomised to sorafenib did not receive their full planned courses, and therefore may have not received a full therapeutic dose thus reducing estimates of effectiveness. FLT3 mutation status may also have affected outcomes.

In our review, FLT3 mutation was not assessed in relation to OS due to lack of data; however, a recent meta-analysis which included non-randomised data has concluded that patients with FLT3-ITD-positive mutations are more sensitive to FLT3 inhibitor treatment, thereby achieving a better CR (OR = 1.89, 95% CI 1.06 to 3.37, *p*= 0.03) and ORR (OR = 3.07, 95% CI 2.13 to 4.43, *p* < 0.001) [26].

The mechanism of action of the agents used may also have affected the findings of this review. Six of the completed trials involved first-generation FLT3 inhibitors—sorafenib, lestaurtinib and midostaurin—and two involved second-generation FLT3 inhibitors—gilteritinib and quizartinib. First-generation inhibitors lack specificity to FLT3 and are therefore not as potent as second-generation FLT3 inhibitors which have been designed to only target FLT3. However, first-generation FLT3 inhibitors can target downstream of FLT3 and may also be effective in parallel signalling pathways and in other targets in AML cells [27]. This may enhance their anti-leukaemic efficacy, particularly in patients who do not have FLT3 mutations, as was the case for a proportion of patients in the older trials. FLT3 inhibitors are also classed as type I and type II. Lestaurtinib, midostaurin and gilteritinib are type I inhibitors whereas sorafenib and quizartinib are type II inhibitors. Type I and II inhibitors differ in the way they interact with the ATP binding site [27]. The consequence being that type I inhibitors can bind to both ITD and TKD mutations, whereas type II inhibitors can only inhibit ITD mutations, with resistance to type II inhibitors occurring when TKD mutations develop. There are nine ongoing trials investigating second-generation FLT3 inhibitors, with eight utilising type I inhibitors (crenolanib—3 trials, gilteritinib—5 trials) and one investigating a type II inhibitor (quizartinib—1 trial), which may eventually show a difference in effect size compared to first-generation inhibitors.

First-generation FLT3 inhibitors have been associated with toxic effects due to their off-target activity. There were broadly similar levels of AEs in both the intervention and control groups across most of the AEs reported for the first-generation inhibitors. As most of the trial participants had previously or were concurrently receiving chemotherapy, it is likely that many of the AEs reported were as a consequence to this.

For second-generation inhibitors, concerns have been raised about cardiac events, particularly an increase in the QT interval, which can lead to cardiac arrhythmia and sudden death. The QuANTUM-R study reported ten patients in the quizartinib arm who had had a grade 3 prolonged QT interval episode which was reported within the adverse events table, whereas episodes of the prolonged QT interval within the ADMIRAL trial were reported within the text, with 12 patients showing a prolonged QT interval, of which one was a QT greater than 501 ms and 6 were greater than 60 ms, which equates to grades 3 and 4 [28]. This example demonstrates how challenging it is to extract data on AEs within these publications, with all the trials reporting either different AEs, or the same events in different formats making an accurate and valid comparison very difficult. Of the second-generation inhibitors, only the ADMIRAL trial (gilteritinib) reported 30- and 60-day mortality, with a substantial benefit in the gilteritinib arm, although patient numbers were relatively low. QuANTUM-R did not report 30- and 60-day mortality but reported ‘treatment-emergent deaths’, of which 80 (33%) were attributable to quizartinib and 16 (17%) to the control treatment. This equates to a RR of 1.93 (95% CI 1.17 to 3.17, *p* = 0.009), which is a substantial difference; however, they did not define ‘treatment-emergent’; therefore, this was not inputted into the pooled early death/mortality analysis.

Only the ADMIRAL trial reported quality of life data, and this was as a conference abstract; therefore, it is difficult to establish the wider implications of the treatment, particularly in this group of life-limited patients.

### Strengths and limitations

Following a systematic methodology and comprehensive search strategy, eight completed RCTs were included in the review for full analysis. Trial publications, conference abstracts and protocols were utilised to augment the comprehensiveness of the review; despite this the review was limited by poor reporting and missing data. Where survival data was not explicitly reported, it was extracted from graphs and/or calculated, which is more imprecise than reported HRs, CIs and *p* values and may introduce error. Additionally, subgroup analyses were also restricted, with some subgroup estimates based solely on the results of a single trial, or not being possible.

Moderate to substantial heterogeneity was found within the pooled analysis. As a fixed-effects model was used, there could be room for criticism; however, the fixed-effect meta-analysis was planned a priori to the findings of the meta-analysis. Whilst there remains debate as to which model is best, the fixed-effect model has its advantages. Firstly, it does not give as much weight to the smaller trials as the random-effects model does, which is beneficial as there is evidence that small trials have a tendency to overestimate treatment effects; using the fixed-effect model can help to mitigate this overestimation. Secondly, although the random-effects model assumes that trials are not linked, prior trials will influence new trial designs. In fixed-effects, the model does not actually assume fixed-effect, but is statistically assumption free; therefore, the ‘assumption-free model’ might be a better term.

### Implications for practice

Midostaurin was approved by the FDA in April 2017 [7] and by the European Medicines Agency (EMA) in September 2017 [29] for patients with newly diagnosed FLT3-mutated AML on the basis of the Stone trial [19]. The marketing authorisation states that patients can be treated with midostaurin as part of induction therapy beginning on day 8 [30]; therefore, this has implications for practice as patients will need early and rapid testing for FLT3-mutation status. Lestaurtinib is no longer in clinical development [27] and sorafenib remains unlicensed for patients with AML. In November 2018, the FDA approved gilteritinib on the basis of interim data (response data and blood transfusion requirements) from the ADMIRAL trial (NCT02421939) [8] in relapsed and refractory patients, whereas quizartinib has been rejected by both the FDA and EMA for marketing authorisation for various reasons such as dropouts (23% of the control group did not receive chemotherapy), censoring and concerns about cardiac and infection AEs [20].

### Implications for future research

There are questions remaining, particularly regarding when to give FLT3 inhibitors and what the effect of prior treatment with FLT3 inhibitor will be on subsequent FLT3 treatment. The completed trials administered FLT3 inhibitors during induction, consolidation and maintenance, and several ongoing trials are administering FLT3 inhibitors after SCT. This suggests that currently there is no evidence regarding when best to use these novel agents in the treatment pathway. In the relapsed and refractory populations, the majority of patients were FLT3 naïve; however, with midostaurin now approved for the treatment of newly diagnosed patients, this will change in future trial cohorts.

We identified 19 ongoing trials, aiming to recruit approximately 8500 patients. The success of these trials may be limited by the number of patients they are able to recruit, given the increasing number of new treatments requiring testing in AML. It may be that novel trial designs such as multi-arm, multi-stage platform trials need to be employed (see NCT01371981 and NCT03164057) in order to maximise resources.

## Conclusion

There is evidence to support the use of FLT3 inhibitors in patients with AML in both the newly diagnosed and relapsed and refractory setting, although there remain questions regarding the optimum use of the drugs and better understanding of the toxicity profile and quality of life outcomes. Currently, this is an active area of research and this review is not a fait accompli; therefore, it is advised that this review is updated once ongoing research has been completed; however, future trials should endeavour to improve reporting to make trial results more transparent and accessible, in order that clinicians can differentiate between treatments to maximise patient benefit.

## Supplementary Information

**Additional file 1. **MEDLINE (Ovid) search strategy for FLT3 inhibitors in patients with AML. **Table S1.** Ongoing trials meeting review inclusion criteria (ordered alphabetically by FLT3 inhibitor [column name: FLT3 inhibitor group details]. **Table S2.** Conference abstracts of trials meeting review inclusion criteria (ordered alphabetically by intervention)*. **Table S3.** Characteristics of included studies. **Table S4.** Treatment schedules for included trials. **Table S5.** Outcome definitions according to trials included in review. **Table S6.** Data sources and methods of calculation for all included outcomes. **Table S7.** Baseline and outcome data for FLT3-mutated patients from Rollig C, 2015. **Table S8.** Baseline and outcome data for FLT3-mutated patients from Serve H, 2013. **Table S9.** Baseline data for FLT3-mutated patients from Knapper S, 2017. **Table S10.** Outcome data for FLT3-mutated patients from Knapper S, 2017. **Table S11.** Baseline and outcome data for FLT3-mutated patients from Levis M, 2011. **Table S12.** Baseline and outcome data for FLT3-mutated patients from Stone RM, 2017. **Table S13.** Baseline and outcome data for FLT3-mutated patients from Perl AE, 2019. **Table S14.** Baseline data from FLT3-ITD-mutated patients from Cortes JE, 2019. **Table S15.** Outcome data for FLT3-mutated AML patients from Cortes JE, 2019. **Fig. S1.** Funnel plot of all eight included trials for overall survival. **Fig. S2.** Forest plot of event-free survival data (uncensored population), grouped by FLT3 inhibitor. **Fig. S3.** Forest plot of relapse-free survival data (uncensored population), grouped by FLT3 inhibitor. **Fig. S4** Forest plot of complete remission data, grouped by FLT3 inhibitor. **Fig. S5.** Forest plot of overall response rate (CR + CRi/CRp) data, grouped by FLT3 inhibitor. **Fig. S6.** Forest plot of overall survival data, grouped by disease stage **Fig. S7.** Forest plot of event-free survival (calculated and reported data), grouped by disease stage. **Fig. S8.** Forest plot of overall survival, grouped by age categories. **Fig. S9.** Forest plot of event-free survival (calculated and reported data), grouped by age categories. **Fig. S10.** Forest plot of overall survival data (censored population), grouped by FLT3 inhibitor. **Fig. S11.** Forest plot of early death/mortality data. **Fig. S12**. Forest plot of vascular adverse events data. **Fig. S13.** Forest plot of dermatological adverse events data. **Fig. S14.** Forest plot of respiratory adverse events data. **Fig. S15.** Forest plot of liver/hepatobiliary adverse events data. **Fig. S16.** Forest plot of haematological adverse events data. **Fig. S17.** Forest plot of metabolism and nutritional disorders data. **Fig. S18.** Forest plot of gastrointestinal disorders data. **Fig. S19.** Forest plot of constitutional symptoms data. **Fig. S20.** Risk of bias graph of included trials. **Fig. S21.** Risk of bias summary of included trials.

## Data Availability

The datasets used and/or analysed during the current study are available from the corresponding author on reasonable request.

## Abbreviations

AE: Adverse event
AML: Acute myeloid leukaemia
ASH: American Society for Hematology
CI: Confidence interval
CR: Complete remission
CRi: Complete remission with incomplete recovery
CRp: Complete remission with incomplete platelet recovery
CTC: Common Toxicity Criteria (National Cancer Institute)
EFS: Event-free survival
EMA: European Medicines Agency
FDA: U.S. Food and Drug Administration
FLT3: FMS-like tyrosine kinase 3
HR: Hazard ratio
ITD: Internal tandem duplication
ORR: Overall response rate
OS: Overall survival
PRISMA: Preferred Reporting Items for Systematic Reviews and Meta-Analyses
RCT: Randomised controlled trial
RFS: Relapse-free survival
RR: Risk ratio
SCT: Stem cell transplant
TKD: Tyrosine kinase domain
