# Supplementary material for: FLT3 inhibitors in acute myeloid leukaemia: assessment of clinical effectiveness, adverse events and future research—a systematic review and meta-analysis
Source: Syst Rev. 2020 Dec 7;9:285. doi: 10.1186/s13643-020-01540-1 (PMC7722339; doi:10.1186/s13643-020-01540-1)
Supplement: Supplementary file 1 — Additional file 1. MEDLINE (Ovid) search strategy for FLT3 inhibitors in patients with AML. Table S1. Ongoing trials meeting review inclusion criteria (ordered alphabetically by FLT3 inhibitor [column name: FLT3 inhibitor group details]. Table S2. Conference abstracts of trials meeting review inclusion criteria (ordered alphabetically by intervention)*. Table S3. Characteristics of included studies. Table S4. Treatment schedules for included trials. Table S5. Outcome definitions according to trials included in review. Table S6. Data sources and methods of calculation for all included outcomes. Table S7. Baseline and outcome data for FLT3-mutated patients from Rollig C, 2015. Table S8. Baseline and outcome data for FLT3-mutated patients from Serve H, 2013. Table S9. Baseline data for FLT3-mutated patients from Knapper S, 2017. Table S10. Outcome data for FLT3-mutated patients from Knapper S, 2017. Table S11. Baseline and outcome data for FLT3-mutated patients from Levis M, 2011. Table S12. Baseline and outcome data for FLT3-mutated patients from Stone RM, 2017. Table S13. Baseline and outcome data for FLT3-mutated patients from Perl AE, 2019. Table S14. Baseline data from FLT3-ITD-mutated patients from Cortes JE, 2019. Table S15. Outcome data for FLT3-mutated AML patients from Cortes JE, 2019. Fig. S1. Funnel plot of all eight included trials for overall survival. Fig. S2. Forest plot of event-free survival data (uncensored population), grouped by FLT3 inhibitor. Fig. S3. Forest plot of relapse-free survival data (uncensored population), grouped by FLT3 inhibitor. Fig. S4 Forest plot of complete remission data, grouped by FLT3 inhibitor. Fig. S5. Forest plot of overall response rate (CR + CRi/CRp) data, grouped by FLT3 inhibitor. Fig. S6. Forest plot of overall survival data, grouped by disease stage Fig. S7. Forest plot of event-free survival (calculated and reported data), grouped by disease stage. Fig. S8. Forest plot of overall survival, grouped by age categories [file 13643_2020_1540_MOESM1_ESM.docx]

Supplementary File 1: MEDLINE (Ovid) search strategy for FLT3 inhibitors in patients with AML

Database: Ovid MEDLINE(R) <1946 to June Week 1 2017> (Updated July 2020)

Search Strategy:

--------------------------------------------------------------------------------

1 exp Leukemia, Myeloid, Acute/ (48424)

2 acut$.tw. (929997)

3 ((myelo$ or nonlympho$ or granulocytic$ or monocyt$ or megakaryoblast$ or promyelocyt$ or erythroblast$) and (leuk?em$ or leuc$)).tw. (94352)

4 2 and 3 (50750)

5 erythroleuk?em$.tw. (5941)

6 (erythremic$ adj myelos$).tw. (95)

7 exp Leukemia, Myelomonocytic, Acute/ (1349)

8 aml.tw. (22631)

9 4 or 5 or 6 or 7 or 8 (60765)

10 1 or 9 (77308)

11 exp fms-Like Tyrosine Kinase 3/ (2076)

12 FLT3.tw. (3529)

13 FLT-3.tw. (558)

14 (sunitinib or sutent or SU11248 or SU-11248).tw. (3618)

15 (sorafenib or nexavar or BAY-439006 or BAY 43-9006 or BAY439006).tw. (4601)

16 (lestaurtinib or CEP-701 or CEP701 or KT-5555 or KT5555).tw. (104)

17 (midostaurin or PKC-412 or PKC412).tw. (240)

18 (quizartinib or AC-220 or AC220).tw. (77)

19 (crenolanib or CP-868-596 or CP 868-596 or CP 868 596 or CP868596).tw. (25)

20 (ponatinib or iclusig or AP-24534 or AP24534).tw. (237)

21 (tandutinib or MLN518 or MLN-518 or MLN0518 or CT-53518 or CT53518).tw. (38)

22 (gilteritinib or ASP2215 or ASP-2215).tw. (2)

23 (nintedanib or BIBF-1120 or BIBF1120 or intedanib or OFEV or vargatef).tw. (231)

24 (pacritinib or ONX-0803 or ONX0803 or pacritinibum or SB-1518 or SB1518).tw. (31)

25 (fedratinib or SAR-302503 or SAR302503 or TG-101348 or TG101348).tw. (55)

26 (pexidartinib or PLX-3397 or PLX3397).tw. (26)

27 (glesatinib or MGCD-265 or MGCD265).tw. (1)

28 (semaxanib or semoxind or SU-5416 or SU5416).tw. (400)

29 (KW-2449 or KW2449).tw. (12)

30 11 or 12 or 13 or 14 or 15 or 16 or 17 or 18 or 19 or 20 or 21 or 22 or 23 or 24 or 25 or 26 or 27 or 28 or 29 (12281)

31 10 and 30 (2316)

32 randomized controlled trial.pt. (445751)

33 controlled clinical trial.pt. (91693)

34 randomized.ab. (338996)

35 placebo.ab. (167647)

36 drug therapy.fs. (1926350)

37 randomly.ab. (233814)

38 trial.ab. (354262)

39 groups.ab. (1456653)

40 32 or 33 or 34 or 35 or 36 or 37 or 38 or 39 (3677510)

41 31 and 40 (989)

42 limit 41 to (humans and yr="2000 -Current") (974)

***************************

Supplementary Table 1: Ongoing trials meeting review inclusion criteria (ordered alphabetically by FLT3 inhibitor [column name: FLT3 inhibitor group details]

| **Trial ID**  **Phase** | **Population**  **Enrolment/sample size** | **FLT3 inhibitor group details** | **Control group details** | **Outcomes** | **Current status, verification date** | **Study completion date** | **Web address** |
| --- | --- | --- | --- | --- | --- | --- | --- |
| NCT03250338;  **ARO-013**  Phase III | 18-75 yrs, rel/ref FLT3-mutated AML  Estimated enrolment 322 | Crenolanib + salvage chemo | Placebo + salvage chemo | EFS, OS, RFS, CR, MRD, CR rate | Recruiting,  June 2020 | October 2021 | [www.clinicaltrials.gov/ct2/show/NCT03250338](http://www.clinicaltrials.gov/ct2/show/NCT03250338) |
| NCT02298166;  AMLSG 19-13; **ARO-007**  Phase III | Aged ≥18 yrs, rel/ref FLT3-mutated AML  N=9 | Crenolanib + chemo | Placebo + chemo | EFS, OS, CR, CRi, CIR, CID, QoL, ED, HD, tox | Terminated,  April 2020 | March 2020 | [www.ClinicalTrials.gov/show/NCT02298166](http://www.ClinicalTrials.gov/show/NCT02298166) |
| NCT03258931;  **ARO-021**  Phase III | Aged 18-60 yrs, newly diagnosed FLT3-mutated AML  Estimated enrolment 510 | Crenolanib + salvage chemo | Midostaurin + salvage chemo | EFS, OS, RFS, CRc rate, duration of response | Recruiting,  May 2020 | November 2022 | <https://clinicaltrials.gov/ct2/show/NCT03258931> |
| NCT02997202  Phase III | Aged ≥18 yrs, FLT3-ITD AML in 1^st^ CR undergoing allogeneic HCT  N=356 | Gilteritinib | Placebo | RFS, AEs, OS, NRM, EFS, cumulative incidence of: acute GVHD, chronic GVHD & detection of FLT3-ITD MRD | Active, not recruiting,  August 2020 | April 2025 | [www.ClinicalTrials.gov/show/NCT02997202](http://www.ClinicalTrials.gov/show/NCT02997202) |
| NCT02927262  Phase III | Aged ≥18 yrs, FLT3-ITD AML in 1^st^ CR  N=98 | Gilteritinib | Placebo | RFS, OS, EFS, MRD, AEs, pharmacodynamics | Active, not recruiting,  July 2020 | June 2021 | [www.ClinicalTrials.gov/show/NCT02927262](http://www.ClinicalTrials.gov/show/NCT02927262) |
| NCT03182244  Phase III | Aged ≥18 yrs, rel/ref FLT3-mutated AML  Estimated enrolment 318 | Gilteritinib | Salvage chemo | OS, EFS, CR, LFS, CRc, CR, CRp, CRi, CRc rate, transplantation rate, fatigue, AEs, pharmacodynamics | Recruiting,  August 2020 | April 2021 | [www.ClinicalTrials.gov/show/NCT03182244](http://www.ClinicalTrials.gov/show/NCT03182244) |
| NCT02752035  3-arm phase II/III | Aged ≥18 yrs, newly diagnosed FLT3-mutated AML, not eligible for intensive induction chemo  Estimated enrolment 250 | Gilteritinib; gilteritinib + azacitidine | Azacitidine alone | OS, EFS, best response, LFS, duration of remission, fatigue, AEs, pharmacodynamics | Recruiting,  June 2020 | April 2021 | [www.ClinicalTrials.gov/show/NCT02752035](http://www.ClinicalTrials.gov/show/NCT02752035) |
| NCT04293562;  AAML1831; NCI-2020-00546  Phase III | Aged <22 yrs, newly diagnosed de novo AML  Estimated enrolment 1,400 | Gilteritinib  Multiarm trial, gilteritinib given across several arms | Various chemo options including with gemtuzumab | EFS, OS, MRD, proportion of death during protocol therapy, relapse rate, TRM rate, AEs, course duration, length of hospitalisation, time to count recovery | Recruiting,  August 2020 | February 2025 | <https://www.clinicaltrials.gov/ct2/show/NCT04293562> |
| NCT01883362;  **RADIUS**  Phase II | Aged 18-60 yrs, FLT3-ITD-mutated AML & undergone allogeneic HSCT  N=60 | Midostaurin + standard care | Standard care | RFS, DFS, NRM, OS, FLT3-ITD mutation status, safety and tolerability, plasma pharmacokinetics | Completed, April 2019 | April 2018 | [www.ClinicalTrials.gov/show/NCT01883362](http://www.ClinicalTrials.gov/show/NCT01883362) |
| NCT03280030  Phase II | Aged 18-64 yrs, newly diagnosed FLT3-mutated AML  N=105 | Midostaurin + chemo | Placebo + chemo | EFS, OS, CR, CIR, safety, QoL, pharmacokinetics | Active, not recruiting,  July 2020 | September 2020 | <https://clinicaltrials.gov/ct2/show/NCT03280030> |
| NCT03092674  Multi-arm Phase II/III | Aged ≥60 yrs, previously untreated AML or high-risk MDS  Estimated enrolment 1,670 | Midostaurin + azacitidine  Multi-arm trial, midostaurin and azacitidine given in Arm C | Azacitidine | OS, CR, CRi, EFS, RFS, AEs, cytogenetics, remission rates | Suspended,  June 2020 | August 2023 | [www.ClinicalTrials.gov/show/NCT03092674](https://clinicaltrials.gov/show/NCT03092674) |
| NCT03512197  Phase III | Aged ≥18 yrs, newly diagnosed FLT3-mutated AML  N=511 | Midostaurin + chemo | Placebo + chemo | EFS, OS, CR, MRD, DFS, CIR, CID, cytogenetics, pharmacokinetics, QoL | Active, not recruiting,  July 2020 | December 2020 | <https://clinicaltrials.gov/ct2/show/NCT03512197> |
| NCT04385290  Phase II (MAGNOLIA);  Phase II (MAGMA) | Aged 18-75 yrs, newly diagnosed AML, FLT3 wild-type (MAGNOLIA), FLT3-ITD or FLT3-TKD (MAGMA)  Estimated enrolment 214 | MAGNOLIA: midostaurin + gemtuzumab ozogamicin + chemo  MAGMA: midostaurin + gemtuzumab ozogamicin + chemo | MAGNOLIA: placebo + chemo  MAGMA: midostaurin + chemo | MTD of midostaurin and gemtuzumab ozogamicin combination, EFS, CR, remission, relapse, RFS, OS, early mortality, AEs, proportion of allogeneic SCT, CD33 expression of AML blasts | Not yet recruiting,  May 2020 | April 2028 | <https://clinicaltrials.gov/ct2/show/NCT04385290> |
| NCT02665143  Phase I/II | Aged ≥18 yrs, ref or 1^st^ rel AML  Estimated enrolment 60 | Nintedanib + induction chemo | Placebo + induction chemo | Phase II: CR rate, incidence of haematological improvement | Suspended,  July 2020 | November 2020 | [www.ClinicalTrials.gov/show/NCT02665143](http://www.ClinicalTrials.gov/show/NCT02665143) |
| ACTRN12614000810617  Phase I/II | Aged ≥18 yrs & failing prior therapy for FLT3-ITD positive AML  Estimated enrolment 60 | Ponatinib | Ponatinib + azacitidine | Phase II: CR, CRi, PR, relapse, QoL, PFS, OS, proportion bridged to allogeneic SCT, time to best response, duration of response | Recruiting,  April 2019 | NR | [www.anzctr.org.au/ACTRN12614000810617.aspx](http://www.anzctr.org.au/ACTRN12614000810617.aspx) |
| NCT02668653;  **QuANTUM-First**  JAPIC CTI 173667  Phase III | Aged 18-75 yrs, newly diagnosed FLT3-ITD AML  N=539 | Quizartinib + chemo | Placebo + chemo | EFS, OS, CR, CRc, % CR with no evidence of MRD | Active, not recruiting,  May 2020 | April 2022 | [www.ClinicalTrials.gov/show/NCT02668653](http://www.ClinicalTrials.gov/show/NCT02668653) |
| ACTRN12611001112954  Phase II | Aged 15-65 yrs, previously untreated FLT3-ITD AML  N=102 | Sorafenib + intensive chemo | Placebo + intensive chemo | EFS, CR, CRi, RFS from CR, TTR, OS, tox, biomarkers of response | Active, not recruiting,  April 2020 | NR | [www.anzctr.org.au/ACTRN12611001112954.aspx](http://www.anzctr.org.au/ACTRN12611001112954.aspx) |
| NCT01371981;  AAML1031;  COG-AAML1031  Multi-arm  Phase III | Aged <29 yrs, newly diagnosed AML. Pts eligible for sorafenib if FLT3-ITD positive  N=1645 | Sorafenib  Multi-arm trial, sorafenib given in Arm C for induction & intensive treatments | Multi-arm trial: 3 comparative regimens | EFS, OS, relapse rate, AEs, TRM, parent-reported questionnaire scores, systemic exposure of sorafenib tosylate and N-oxide metabolite for each course of induction & intensification | Active, not recruiting,  July 2020 | March 2019 | <https://ClinicalTrials.gov/show/NCT01371981> |
| NCT03164057;  **AML-16**  Multi-arm  Phase II | Aged <21 yrs, newly diagnosed AML  Estimated enrolment 200 | Sorafenib  Multi-arm trial, 8 regimes with sorafenib | Multi-arm trial: 16 regimes without sorafenib | OS, EFS, proportion of evaluable patients who tolerate 5 days of single agent DMTi before standard chemo combination, change in genome-wide methylation burden of leukaemia, MRD-evaluable subjects | Recruiting, June 2020 | June 2025 | <https://ClinicalTrials.gov/show/NCT03164057> |

Abbreviations: AEs, adverse events; AML, acute myeloid leukaemia; chemo, chemotherapy; CID, cumulative incidence of death; CIR, cumulative incidence of relapse; CR, complete remission; CRc, composite complete remission; CRi, complete remission with incomplete hematologic recovery; CRp, complete remission with incomplete platelet recovery; DFS, disease-free survival; DMTi, DNA methyltransferase inhibitor; ED, early death; EFS, event-free survival; FLT3, fms-like tyrosine kinase 3; FLT3-ITD, FLT3-internal tandem duplication; FLT3-TKD, FLT3-tyrosine kinase domain; GVHD, graft versus host disease; HD, hypoplastic death; HCT, hematopoietic cell transplant; HSCT, hematopoietic stem cell transplant; LFS, leukaemia-free survival; MDS, myelodysplastic syndrome; MRD, minimal residual disease; NR, not reported; NRM, non-relapse mortality; OS, overall survival; PFS, progression-free survival; PR, partial response; pts, patients; QoL, quality of life; ref, refractory; rel, relapsed; RFS, relapse-free survival; SCT, stem cell transplant; tox, toxicity; TRM, treatment-related mortality; TTR, time-to-relapse; yrs, years.

Supplementary Table 2: Conference abstracts of trials meeting review inclusion criteria (ordered alphabetically by intervention)*

| **No.** | **Trial ID & abstract details** | **Authors** | **Title** | **Population** | **Intervention & comparator/s** | **Outcomes/Results** |
| --- | --- | --- | --- | --- | --- | --- |
| 1 | [NCT03258931](http://clinicaltrials.gov/show/NCT03258931);  Phase III  ASCO, 2019  Protocol | Stone RM, Wang ES, Goldberg AD, Sweet KL, Fathi AT, Liu H, *et al* | Crenolanib versus midostaurin combined with induction and consolidation chemotherapy in newly diagnosed FLT3 mutated AML | Adults aged 18-60 yrs, newly diagnosed FLT3-mutated AML, eligible for intensive chemotherapy  Estimated enrolment 510 | Crenolanib vs midostaurin, combined with intensive chemotherapy | EFS  <https://meetinglibrary.asco.org/record/178067/abstract> |
| 2 | NCT02752035  Phase II/III  ASCO, 2017  Protocol | Cortes JE, Altman J, Ritchie EK, Larson RA, Claxton D, Minden MD, *et al* | A phase II/III, multicenter, open-label, 3-arm study of gilteritinib, gilteritinib plus azacitidine, or azacitidine alone in the treatment of newly diagnosed FLT3 mutation-positive acute myeloid leukemia (AML) patients ineligible for intensive induction chemotherapy | Newly diagnosed FLT3-mutated AML  Estimated enrolment 323 | Gilteritinib vs azacitidine plus gilteritinib vs azacitidine | OS, EFS, CR rate, CRc rate, LFS, remission duration, tolerability, fatigue  <http://ascopubs.org/doi/abs/10.1200/JCO.2017.35.15_suppl.TPS7068> |
| 3 | NCT02997202  Phase III  ASCO, 2018  Protocol | Mark J. Levis, Mehdi Hamadani, Brent Logan, Matt Rosales, Alexander E. Perl, Steven Michael Devine, *et al* | A phase 3, trial of gilteritinib, as maintenance therapy after allogeneic hematopoietic stem cell transplantation in patients with FLT3-ITD+ AML | Aged ≥18 yrs, FLT3-ITD+ AML in 1^st^ CR who are ≥30 days & ≤90 days from scheduled allogeneic HSCT  Estimated enrolment 346 | Gilteritinib vs placebo | RFS, OS, safety, NRM, EFS, incidence of acute/chronic GVHD, MRD  <http://ascopubs.org/doi/abs/10.1200/JCO.2018.36.15_suppl.TPS7075> |
| 4a | [NCT01883362](http://www.bloodjournal.org/lookup/external-ref?link_type=CLINTRIALGOV&access_num=NCT01883362&atom=%2Fbloodjournal%2F132%2FSuppl_1%2F662.atom);  **RADIUS**  Phase II  ASH, 2018  Primary results | Richard Thomas T. Maziarz, Mrinal M. Patnaik, Bart L Scott, Sanjay R. Mohan, Abhinav Deol, Scott D. Rowley, *et al* | Radius: A Phase 2 Randomized Trial Investigating Standard of Care ± Midostaurin after Allogeneic Stem Cell Transplant in FLT3-ITD-Mutated AML | Aged 18-70 yrs, FLT3-mutated AML & had undergone SCT in 1^st^ CR  N=60 | Midostaurin plus standard care vs standard care | Int (n=30) vs cont (n=30):  18 mth RFS, 89% vs 76%;  Estimated relapse rates, 11% vs 24%;  AEs, 100% vs 87%;  any grade vomiting, 73% vs 23%;  serious AEs, 30% vs 57% (diarrhoea, nausea, vomiting, pyrexia most common)  8 pts discontinued midostaurin due to AEs (mostly gastrointestinal);  Deaths, 4 vs 8  <https://ash.confex.com/ash/2018/webprogram/Paper113582.html> |
| 4b | NCT01883362;  **RADIUS**  Phase II  ASCO, 2015  Protocol | Maziarz RT, Scott BL, Mohan SR, Deol A, Patnaik MM, Haines K, *et al* | A phase II, randomized trial of standard of care with or without midostaurin to prevent relapse following allogeneic stem cell transplantation in patients with FLT3-ITD mutated acute myeloid leukemia | Aged 18-60 yrs, FLT3-mutated AML  Estimated enrolment 60 pts | Midostaurin plus standard care vs standard care | Relapse at 18 mths post-HSCT, DFS, NRM, OS, safety, pharmacokinetics  <http://ascopubs.org/doi/abs/10.1200/jco.2015.33.15_suppl.tps7094> |
| 5a | NCT02668653;  **QuANTUM-First**  Phase III  ESMO, 2017  Protocol | R. Schlenk, H. Dombret, S. Amadori, P. Montesinos, M. Levis, M.A. Sekeres, *et al* | QuANTUM-First: phase 3, double-blind, placebo-controlled study of  quizartinib in combination with induction and consolidation chemotherapy, and as maintenance therapy in patients (pts) with newly diagnosed (NDx) FLT3-ITD acute myeloid leukemia (AML) | Aged 18-75 yrs, newly diagnosed FLT3-ITD AML  Estimated enrolment 536 | Quizartinib plus chemotherapy vs placebo plus chemotherapy | EFS, OS, CR, CRc  <https://oncologypro.esmo.org/Meeting-Resources/ESMO-2017-Congress/QuANTUM-First-phase-3-double-blind-placebo-controlled-study-of-quizartinib-in-combination-with-induction-and-consolidation-chemotherapy-and-as-maintenance-therapy-in-patients-pts-with-newly-diagnosed-NDx-FLT3-ITD-acute-myeloid-leukemia-AML> |
| 5b | NCT02668653;  **QuANTUM-First**  Phase III  ASCO, 2016  Protocol | Erba HP, Levis MJ, Sekeres MA, Dombret H, Amadori S, Zernovak O, Edward D, *et al* | Phase 3 (P3) study of quizartinib (Q) or placebo (P) with induction (IND) and consolidation chemotherapy (CON) and as maintenance (MN) in patients (pts) with newly diagnosed (NDx) FLT3-ITD–positive acute myeloid leukemia (AML): the QuANTUM-First study | Aged 18-75 yrs, newly diagnosed FLT3-ITD AML  Estimated enrolment 536 | Quizartinib plus chemotherapy vs placebo plus chemotherapy | EFS, OS, CR rate, CRc rate, MRD  <http://ascopubs.org/doi/abs/10.1200/JCO.2016.34.15_suppl.TPS7073> |
| 6 | EudraCT 2010-018539-16;  **SORMAIN**  ASH, 2018  Results | Andreas Burchert, Gesine Bug, Jürgen Finke, Matthias Stelljes, Christoph Rollig, Ralph Wäsch, *et al* | Sorafenib As Maintenance Therapy Post Allogeneic Stem Cell Transplantation for FLT3-ITD Positive AML: Results from the Randomized, Double-Blind, Placebo-Controlled Multicentre Sormain Trial | Aged ≥18 yrs, FLT3-mutated AML & had undergone SCT  N=83 | Sorafenib vs placebo | Int (n=43) vs cont (n=40):  Median RFS, not reached vs 30.9mths;  2-yr RFS, 85.0% vs 53.3%, HR=0.39, p=0.0139  <https://ash.confex.com/ash/2018/webprogram/Paper112614.html> |

*The following five conference proceedings were searched from 2014 to July 2020: ASCO, ASH, BSH, EHA and ESMO. No conference abstracts were identified from BSH.

Abbreviations: AEs, adverse events; AML, acute myeloid leukaemia; ASCO, American Society of Clinical Oncology; ASH, American Society of Hematology; BSH, British Society for Haematology; CIR, cumulative incidence of relapse; cont, control; CR, complete remission; CRc, composite complete remission; DFS, disease-free survival; EFS, event-free survival; EHA, The European Hematology Association; ESMO, European Society for Medical Oncology; FLT3, fms-like tyrosine kinase 3; FLT3-ITD, FLT3-internal tandem duplication; GVHD, graft versus host disease; HR, hazard ratio; HSCT, hematopoietic stem cell transplant; int, intervention; LFS, leukaemia-free survival; MRD, minimal residual disease; mths, months; NRM, non-relapse mortality; OS, overall survival; pts, patients; RFS, relapse-free survival; SCT, stem cell transplant; vs, versus; yr/s, years.

Supplementary Table 3: Characteristics of included studies

| **Study details** | **FLT3 inhibitor group** | **Control group** | **Inclusion /exclusion criteria** | **Age**  **median (range)** | **Sex**  **% male** | **Disease classification** | | | **Cytogenetics**  **%** |
| --- | --- | --- | --- | --- | --- | --- | --- | --- | --- |
|  |  |  |  |  |  | **Type of AML**  **%** | **FLT3 mutational status**  **%** | **NPM1 status %** |  |
| **Rollig C, 2015**  Trial ID: **SORAML**, Study Alliance Leukemia, NCT00893373 Phase 2, multi-institutional RCT in Germany  Follow-up: 36 mths (median)  N=276  (9 untreated), 267 included in analyses | Sorafenib 400mg, twice daily + standard chemo  N=138  (4 untreated), 134 included in analyses | Placebo + standard chemo  N=138  (5 untreated), 133 included in analyses | Aged 18-60 yrs, newly diagnosed de novo or secondary AML (excluding APL) | INT: 50 (43-56)  CONT: 50 (44-55) | INT: 53  CONT: 47 | *Secondary AML*  INT: 10  CONT: 15 | INT: 17  *Missing,* 1  CONT: 17  *Missing,* 0 | INT: 32 *Missing,* 3  CONT: 32  *Missing,* 1 | *Low risk*  INT: 10  CONT: 8  *Intermediate risk*  INT: 66  CONT: 67  *High-risk*  INT: 17  CONT: 20  *Normal Karyotype*  INT: 51  CONT: 50  *Not assessable*  INT: 7  CONT: 5 |
| **Serve H, 2013**  Study Alliance Leukemia  Trial ID: NCT00373373 Phase 2, multi-institutional RCT in Germany  Follow-up: 29.3 mths (median)  N=201,  197 included in analyses | Sorafenib 400mg, twice daily + intensive chemo  N=104,  102 included in analyses | Placebo + intensive chemo  N=97,  95 included in analyses | Aged >60 yrs, de novo or secondary AML or AML from MDS (excluding FAB type M3). | INT: 67.5 (61-78)  CONT: 69 (61-80) | INT: 61  CONT: 53 | *De novo:*  INT: 60  CONT: 61  *Secondary:*  INT: 40  CONT: 39 | *FLT3-ITD alone*  INT: 15  CONT: 14 | INT: 21  CONT: 19 | *Unfavourable karyotype*  INT: 19  CONT: 21 |
| **Knapper S, 2017**  **(from AML 15)**  Trial ID: ISRCTN17161961  Phase 3, multicentre RCT in UK, Denmark, New Zealand  Follow-up: 50.5 mths (median)  N=175 | Lestaurtinib 80mg, twice daily administered after each of 4 courses of intensive chemo  N=88 | Intensive chemo only  N=87 | Aged <60 yrs, de novo or secondary AML (excluding APL), FLT3 mutation | INT: 48 (16-66)  CONT: 46 (16-65) | INT: 47  CONT: 41 | *De novo*  INT: 95  CONT: 97  *Secondary*  INT: 3  CONT: 5  *High-risk MDS*  INT: 0  CONT: 0 | *FLT3-ITD alone*  INT: 74  CONT: 75  *FLT3-TKD alone*  INT: 25  CONT: 21  *FLT3-ITD+TKD* INT: 1  CONT: 2  *Not assessable* INT: 0  CONT: 2 | *Wildtype*  INT: 48 CONT: 39  *Mutant*  INT: 49  CONT: 52  *Not known* INT: 3  CONT: 9 | *Favourable*  INT: 6  CONT: 7  *Intermediate*  INT: 73  CONT: 79  *Adverse*  INT: 8  CONT: 6  *Unknown*  INT: 14  CONT: 8 |
| **Knapper S, 2017**  **(from AML 17)**  Trial ID: ISRCTN55675535  Phase 3, multicentre RCT in UK, Denmark, New Zealand  Follow-up: 50.5 mths (median)  N=325 | Lestaurtinib 80mg, twice daily + 1^st^ line intensive chemo  N=212 | Placebo + 1^st^ line intensive chemo  N=113 | Aged <60 yrs, de novo or secondary AML (excluding APL), FLT3 mutation | INT: 50  (5-68)  CONT: 50 (6-65) | INT: 47  CONT: 50 | *De novo*  INT: 93  CONT: 92  *Secondary*  INT: 5  CONT: 5  *High-risk* *MDS*  INT: 2  CONT: 3 | *FLT3-ITD alone*  INT: 73  CONT: 75  *FLT3-TKD alone*  INT: 25  CONT: 20  *FLT3-ITD+TKD* INT: 2  CONT: 4  *Not assessable* INT: 0.5  CONT: 1 | *Wildtype*  INT: 20  CONT: 30  *Mutant*  INT: 20  CONT: 40  *Not known* INT: 1  CONT: 7 | *Favourable*  INT: 5  CONT: 4  *Intermediate*  INT: 90  CONT: 85  *Adverse*  INT: 3  CONT: 4  *Unknown*  INT: 2  CONT: 5 |
| **Levis M, 2011**  Trial ID:  **Cephalon-204**, NCT00079482  Phase 2 multinational RCT in Australia, Canada, EU, Israel, New Zealand, Russian Federation, Ukraine, USA.  Follow-up: NR  N=224 | Lestaurtinib 80mg, twice daily + salvage chemo  N=112 | Salvage chemo alone  N=112 | Aged ≥18 yrs, AML with 1^st^ relapse after 1^st^ remission of 1-24 mths, FLT3 mutation | INT: 59 (20-81)  CONT: 54 (21-79) | INT: 45  CONT: 47 | *FAB type*  INT:  M0, 4  M1, 6  M2, 12  M4, 18  M5, 9  M6, 0  M7, 0  Unknown, 52  CONT:  M0, 2  M1, 13  M2, 13  M4, 23  M5, 5  M6, 0  M7, 0  Unknown, 44 | *FLT3-ITD alone*  INT: 101  CONT: 97  *D835 alone* INT: 9  CONT: 8  *ITD and D835*  INT: 2  CONT: 6  *Not confirmed* INT: 0  CONT: 1 | NR | NR |
| **Stone RM, 2017**  Trial ID: **RATIFY** calgb 10603, NCT00651261  Phase 3, mulitcentre RCT in Canada, USA  Follow-up:  59 mths (median)  N=717 | Midostaurin 50mg, twice daily + standard chemo  N=360 | Placebo + standard chemo  N=357 | Aged 18-59 yrs, newly diagnosed AML (excluding APL), FLT3 mutation | INT: 47.1 (19-60)  CONT: 48.6  (18-61) | INT: 48  CONT: 41 | NR | *FLT3-ITD low*  INT: 48  CONT: 48  *FLT3-ITD high*  INT: 30  CONT: 30  *FLT3-TKD*  INT: 23  CONT: 23 | NR | *Favourable*  INT: 6  CONT: 5  *Normal*  INT: 64  CONT: 73  *Intermediate II*  INT: 22  CONT: 16  *Adverse*  INT: 8  CONT: 6 |
| **Perl AE, 2019**  Trial ID: **ADMIRAL,** NCT02421939  Phase 3,  muticentre RCT in 14 countries  Follow-up: 17.8 mths (median)  N=371 | Gilteritinib 120mg, once daily  N=247 | Salvage chemo  N=124 | Aged >18 yrs, relapsed or refractory AML, FLT3 mutation | INT: 62.0 (19.0-85.0)  CONT: 61.5 (19.0-85.0) | INT: 47  CONT: 43.5 | NR | *FLT3-ITD only*  INT: 87.0  CONT: 91.1  *FLT3-TKD only*  INT: 8.5  CONT: 8.1  *FLT3-ITD and TKD*  INT: 2.8  CONT: 0 | NR | *Favourable*  INT: 1.6  CONT: 0.8  *Intermediate*  INT: 73.7  CONT: 71.8  *Unfavourable*  INT: 10.5  CONT: 8.9  *Unknown*  INT: 15.6  CONT: 18.5 |
| **Cortes JE, 2019**  Trial ID: **QuANTUM-R,**  NCT02039726  Phase 3, multicentre RCT in 19 countries  Follow-up: 23.5 mths (median)  N=367 | Quizartinib 20-60mg as appropriate, once daily  N=245 | Salvage chemo  N=122 | Aged >18 yrs, relapsed or refractory AML, (excluding APL), FLT3-ITD mutation | INT: 55.0 (46.0-65.0)  CONT: 57.5 (44.0-66.0) | INT: 46  CONT: 52 | NR | *FLT3-ITD*  100 | INT: 47  CONT: 47 | *Favourable*  INT: 5  CONT: 7  *Intermediate*  INT: 78  CONT: 66  *Unfavourable*  INT: 9  CONT: 11  *Unknown*  INT: 8  CONT: 16 |

Abbreviations: AML, acute myeloid leukaemia; APL, acute promyelocytic leukaemia; chemo, chemotherapy; CONT, control; FAB, French-American British (AML classification system); FLT3, fms-like tyrosine kinase 3; FLT3-ITD, FLT3-internal tandem duplication; FLT3-TKD, FLT3-tyrosine kinase domain; INT, intervention; MDS, myelodysplastic syndrome; mths, months; NPM1, nucleophosmin 1; NR, not reported; RCT, randomised controlled trial; yrs, years.

Supplementary Table 4: Treatment schedules for included trials

| **Author, year** | **Intervention** | **Control** | **1st Induction** | **2nd Induction** | **1st Consolidation** | **2nd Consolidation** | **Maintenance** |
| --- | --- | --- | --- | --- | --- | --- | --- |
| Serve H, 2013 | Sorafenib 400mg, twice daily from 3 days post chemo to 3 days pre subsequent chemo course | 2 placebo tablets, twice daily | Cytarabine 100mg/m^2^, days 1-7; daunorubicin 60mg/m^2^, days 3-5 | Cytarabine 100mg/m^2^ for 7 days; daunorubicin 60mg/m^2^ for 3 days. Int or cont as detailed | (2 courses)  cytarabine 1g/m^2^, every 12 hrs on days 1, 3, 5. Int or cont as detailed |  | Int or cont as detailed until 1 yr after start of induction therapy |
| Rollig C, 2015  **(SORAML)** | Sorafenib 400mg, twice daily on days 10-19 | Placebo, twice daily on days 10-19 | Cytarabine 100mg/m^2^ days 1-7; daunorubicin 60mg/m^2^ days 3-5. Int or cont as detailed | Identical to 1^st^ induction with int or cont as detailed | (3 courses) cytarabine 3g/m^2^, 3 hr infusion, twice daily on days 1, 3, 5 followed by int or cont on day 8 until 3 days before next consolidation cycle |  | Int or cont as detailed, continuously for 12 mths after the last consolidation cycle |
| Levis M, 2011  **(Cephalon-204)** | Lestaurtinib 80mg, twice daily (12 hrs between doses) from 2 days post chemo | Chemo only. Cont patients could crossover to lestaurtinib arm if partial response achieved by day 42 marrow assessment | MEC  (Mitoxantrone 8mg/m^2^ daily; etoposide 100mg/m^2^; cytarabine 1000mg/m^2^, days 1-5)  HiDAC  (Cytarabine 1500mg/m^2^, days 1-5) | Identical to 1^st^ induction with int or cont as detailed |  |  |  |
| Knapper S, 2017 **(AML15)*** | Lestaurtinib 80mg, twice daily (12 hrs between doses) from 2 days post chemo to 2 days pre subsequent course, up to a maximum of 28 days, max 4 cycles | Chemo only | ADE 10 + 3 + 5 (Daunorubicin, cytarabine, etoposide); or DA 3 + 10 (Daunorubicin & cytarabine as above); or  FLAG-Ida (Fludarabine, cytarabine, G-CSF [lenograstim], idarubicin). All 3 arms with or without gemtuzumab ozogamicin | ADE 8 + 3 + 5 (Daunorubicin, cytarabine, etoposide); or  DA + 3 (Daunorubicin & cytarabine as above); or  FLAG-Ida (Fludarabine, cytarabine, G-CSF [lenograstim], idarubicin) | MACE  (Amsacrine, cytarabine, etoposide); or  cytarabine 1.5; or  cytarabine 3. All 3 arms with or without gemtuzumab ozogamicin | MidAC (Mitoxantrone, cytarabine); or cytarabine 1.5; or cytarabine 3 | No maintenance therapy with lestaurtinib |
| Knapper S, 2017 **(AML17)*** | Lestaurtinib 40-80mg, twice daily (depending on azole antifungals) from 2 days post chemo to 2 days pre subsequent course, up to a maximum of 28 days | Placebo + chemotherapy | Daunorubicin 90mg/m^2^ days 1, 3, 5 + cytarabine 100mg/m^2^ every 12 hrs days 1-10; or  daunorubicin 60mg/m^2^ + cytarabine 100mg/m^2^ every 12 hrs days 1-10 | Dependent on risk of relapse:  (1) *Core Binding factor (CBF), or CBF (favourable)*  (2) *FLT3 mutant*  (3) *Not CBF, not in CEP-701 rand, not poor risk*  (4) *Poor risk* | Those patients in the CBF, FLT3-mutant and [not CBF, not in CEP-701 rand, not poor risk] arms for the second induction course, who were deemed:  *(1)* *Not poor risk*  *(2)* Poor risk | For details of 2^nd^ consolidation therapy, see 1^st^ consolidation | No maintenance therapy with lestaurtinib |
| Stone RM,  2017  **(RATIFY)** | Midostaurin 50mg, twice daily on days 8-21 | Placebo, twice daily on days 8-21 | Cytarabine 200mg/m^2^, days 1-7; daunorubicin 60mg/m^2^, days 1-3. Midostaurin or control administered on day 8 | Identical to 1^st^ induction and including midostaurin or placebo administration as detailed | (Four 28-day cycles) Cytarabine 3000mg/m^2^, over 3 hrs every 12 hrs on days 1, 3, 5) plus midostaurin or placebo administered as detailed |  | Midostaurin or placebo administered as detailed for twelve 28-days cycles |
| Perl AE, 2019  **(ADMIRAL)** | Gilteritinib 120mg, once daily in 28-day cycles | Salvage chemo, 4 options chosen by local investigator:  MEC (mitoxantrone 8mg/m^2^, etoposide 100mg/m^2^, cytarabine 1000mg/m^2^ once daily on days 1-5);  FLAG-IDA (G-CSF 300mg/m^2^ given on days 1-5 plus fludarabine 30mg/m^2^, cytarabine 2000mg/m^2^ each given once daily on days 2-6, idarubicin 10mg/m^2^ once daily on days 2-4);  LoDAC (cytarabine 20mg twice daily for 10 days);  azacytidine 75mg/m^2^ daily on days 1-7 | | | | | |
| Cortes JE, 2019  **(QuANTUM-R)** | Quizartinib 20-60mg once daily in 28-day cycles | Salvage chemo, 3 options of preselected chemo of investigator’s choice:  LoDAC (low-dose cytarabine 20mg twice daily on days 1-10 of 28-day cycle);  MEC (mitoxantrone 8mg/m^2^/day, etoposide 100mg/m^2^/day, cytarabine 1000mg/m^2^/day on days 1-5 of up to two 28-day cycles);  FLAG-IDA (G-CSF 30mg/m^2^/day on days 2-6, cytarabine IV 2000mg/m^2^/day on days 2-6, idarubicin 10mg/m^2^/day on days 2-4 in up to two 28-day cycles) | | | | | |

*Further dose, duration and chemotherapy combination details can be found in publications related to the original AML15 and AML17 trials.

Abbreviations: chemo, chemotherapy; cont, control; G-CSF, ganulocyte-colony stimulating factor; hrs, hours; int, intervention; mths, months; rand, randomisation; yrs, years.

Supplementary Table 5: Outcome definitions according to trials included in review

| **Study**  **Outcome** | **Knapper S, 2017**  **AML 15** | **Knapper S, 2017**  **AML 17** | **Levis M, 2011** | **Rollig C, 2015** | **Serve H, 2013** | **Stone RM, 2017** | **Perl AE, 2019** | **Cortes JE, 2019** |
| --- | --- | --- | --- | --- | --- | --- | --- | --- |
| Complete remission (CR) | As per revised IWG criteria | As per revised IWG criteria | As per revised IWG criteria | As per revised IWG criteria | As per revised IWG criteria | Presence of <5% blasts in marrow of extramedullary leukaemia, absolute neutrophil count >1,000/µl, platelet count >100,000/µl, & absence of blasts in peripheral blood. Per protocol, CR to have occurred by day 60. | As per the revised International Working Group Criteria (Cheson 2003) | As per the revised International Working Group Criteria (Cheson 2003) |
| Complete response with incomplete recovery (CRi) | As per revised IWG criteria | As per revised IWG criteria | N/A | N/A | NR, although response criteria defined as per revised IWG criteria | N/A | As per the revised International Working Group Criteria (Cheson 2003) | As per the revised International Working Group Criteria (Cheson 2003) |
| Complete response with incomplete platelet recovery (CRp) | N/A | N/A | As per revised IWG criteria | N/A | N/A | N/A | As per the revised International Working Group Criteria (Cheson 2003) | As per the revised International Working Group Criteria (Cheson 2003) |
| Overall response rate (CR + CRi) | As per revised IWG criteria | As per revised IWG criteria | N/A | N/A | NR, although response criteria defined as per revised IWG criteria | N/A | N/A | NR |
| Overall response rate (CR + CRp) | N/A | N/A | As per revised IWG criteria | N/A | N/A | N/A |  |  |
| Overall survival (OS) | As per revised IWG criteria | As per revised IWG criteria | NR | As per revised IWG criteria | As per revised IWG criteria | Time from randomisation to death from any cause | Time from the date of randomisation until the date of death from any cause. For subjects who are not known to have died by the end of study follow-up, OS was censored at the date of last contact | Time from randomisation until death from any cause |
| Event-free survival (EFS) | The earliest of death, relapse or no CR within 61 days of start of induction | The earliest of death, relapse or no CR within 61 days of start of induction | N/A | An event defined as either primary treatment failure or relapse or death. | As per revised IWG criteria | Time from randomisation to earliest qualifying event, including failure to obtain a CR on/before 60 days of initiation of protocol therapy (protocol-specified CR); relapse; or death from any cause | Time from the date of randomisation until the date of documented relapse (excluding relapse after PR), treatment failure, or death, whichever occurs first. For subjects who are not known to have had a relapse, treatment failure, or death event, EFS was censored at the date of last relapse-free disease assessment | Time from randomisation until documented failure to achieve composite CR (CRc), relapse after CRc, or death from any cause, whichever occurred first |
| Relapse-free survival (RFS) | As per revised IWG criteria | As per revised IWG criteria | N/A | Time interval from day 1 of study treatment until relapse from CR | N/A | N/A | N/A | N/A |
| Treatment-related mortality | NR | NR | NR | N/A | N/A | N/A | N/A | N/A |
| Early death | 30- or 60-day mortality | 30- or 60-day mortality | N/A | NR | Death before induction response could be evaluated | N/A | N/A | N/A |
| 30-day mortality | N/A | N/A | Death within 30 days of start of treatment | N/A | N/A | N/A | NR | N/A |
| 60-day mortality | N/A | N/A | N/A | N/A | N/A | N/A | NR | N/A |
| Toxicity  [(Serious) Adverse events] | As per NCI CTC version 3.0 | As per NCI CTC version 3.0 | As per NCI CTC version 3.0 | As per NCI CTC version 3.0 | As per NCI CTC version 3.0 | As per NCI CTC version 3.0 | Safety was assessed by evaluating the incidence of adverse events, including evaluation of vital signs, and results from clinical laboratory tests, electrocardiograms and ophthalmologic examinations | As per NCI CTC version 3.0 |

Abbreviations: CR, complete remission; IWG: International Working Group criteria as per Chesson et al; N/A, not applicable; NCI CTC, National Cancer Institute Common Toxicity Criteria; NR, not reported.

Supplementary Table 6: Data sources and methods of calculation for all included outcomes

| **Outcome** | **FLT3 group events (n)** | **FLT3 group total pts (N)** | **Control group events (n)** | **Control group total pts (N)** | **HR** | **95% CI** | **P-value** | **O-E** | **Variance** | **Method used for calculation** |
| --- | --- | --- | --- | --- | --- | --- | --- | --- | --- | --- |
| **Overall survival (uncensored)** |  |  |  |  |  |  |  |  |  |  |
| Rollig C, 2015 | Cal | ✓ | Cal | ✓ | ✓ | ✓ | ✓ | Cal | Cal | HR & CI |
| Serve H, 2013 | Cal | ✓ | Cal | ✓ | ✓ | ✓ | ✓ | Cal | Cal | HR & CI |
| Knapper S, 2017 (AML15) | ✓ | ✓ | ✓ | ✓ | ✓ | ✓ | ✓ | ✓ | ✓ |  |
| Knapper S, 2017 (AML17) | ✓ | ✓ | ✓ | ✓ | ✓ | ✓ | ✓ | ✓ | ✓ |  |
| Levis M, 2011 | Cal | ✓ | Cal | ✓ | Cal | Cal | n/a | Cal | Cal | Events & pt N |
| Stone RM, 2017 | Cal | ✓ | Cal | ✓ | ✓ | ✓ | ✓ | Cal | Cal | HR & CI |
| Perl AE, 2019 | ✓ | ✓ | ✓ | ✓ | ✓ | ✓ | ✓ | Cal | Cal | CI and HR |
| Cortes JE, 2019 | Cal | ✓ | Cal | ✓ | ✓ | ✓ | ✓ | Cal | Cal | CI and HR |
| **Overall survival (censored)** |  |  |  |  |  |  |  |  |  |  |
| Rollig C, 2015 | n/a | ✓ | n/a | ✓ | ✓ | ✓ | ✓ | Cal | Cal | HR & CI |
| Knapper S, 2017 (AML15) | ✓ | ✓ | ✓ | ✓ | ✓ | ✓ | ✓ | ✓ | ✓ |  |
| Knapper S, 2017 (AML17) | ✓ | ✓ | ✓ | ✓ | ✓ | ✓ | ✓ | ✓ | ✓ |  |
| Levis M, 2011 | Cal | Cal | Cal | Cal | Cal | Cal | Cal | Cal | Cal | Events & pt N |
| Stone RM, 2017 | Cal | ✓ | Cal | ✓ | Cal | Cal | ✓ | Cal | Cal | Events & p-value |
| Perl AE, 2019 | n/a | ✓ | n/a | ✓ | ✓ | ✓ | n/a | Cal | Cal | HR & CI |
| Cortes JE, 2019 | n/a | ✓ | n/a | ✓ | ✓ | ✓ | ✓ | Cal | Cal | HR & CI |
| **Event-free survival (uncensored)*** |  |  |  |  |  |  |  |  |  |  |
| Rollig C, 2015 | Cal | ✓ | Cal | ✓ | ✓ | ✓ | ✓ | Cal | Cal | HR & CI |
| Serve H, 2013 | Cal | ✓ | Cal | ✓ | ✓ | ✓ | ✓ | Cal | Cal | HR & CI |
| Stone RM, 2017 | Cal | ✓ | Cal | ✓ | ✓ | ✓ | ✓ | Cal | Cal | HR & CI |
| Perl AE, 2019 | n/a | ✓ | n/a | ✓ | ✓ | ✓ | n/a | Cal | Cal | HR & CI |
| Cortes JE, 2019 | Cal | ✓ | Cal | ✓ | ✓ | ✓ | ✓ | Cal | Cal | HR & CI |
| **Event-free survival (censored)** |  |  |  |  |  |  |  |  |  |  |
| Rollig C, 2015 | Cal | ✓ | Cal | ✓ | ✓ | ✓ | ✓ | Cal | Cal | HR & CI |
| **Relapse-free survival (uncensored)** |  |  |  |  |  |  |  |  |  |  |
| Rollig C, 2015 | Cal | ✓ | Cal | ✓ | ✓ | ✓ | ✓ | Cal | Cal | HR & CI |
| Knapper S, 2017 (AML15) | ✓ | ✓ | ✓ | ✓ | ✓ | ✓ | ✓ | ✓ | ✓ |  |
| Knapper S, 2017 (AML17) | ✓ | ✓ | ✓ | ✓ | ✓ | ✓ | ✓ | ✓ | ✓ |  |
| Stone RM, 2017 | Cal | ✓ | Cal | ✓ | Cal | Cal | ✓ | Cal | Cal | Events & p-value |
| **Relapse-free survival (censored)** |  |  |  |  |  |  |  |  |  |  |
| Rollig 2015 | n/a | n/a | n/a | n/a | ✓ | ✓ | ✓ | Cal | Cal | HR & CI |
| **Complete remission (CR)** |  |  |  |  |  |  |  |  |  |  |
| Rollig C, 2015 | ✓ | ✓ | ✓ | ✓ |  |  |  |  |  |  |
| Serve H, 2013 | Cal | ✓ | Cal | ✓ |  |  | ✓ |  |  |  |
| Levis M, 2011 | ✓ | ✓ | ✓ | ✓ |  |  | ✓ |  |  |  |
| Stone RM, 2017 | ✓ | ✓ | ✓ | ✓ |  |  | ✓ |  |  |  |
| Perl AE, 2019 | ✓ | ✓ | ✓ | ✓ |  |  |  |  |  |  |
| Cortes JE, 2019 | ✓ | ✓ | ✓ | ✓ |  |  |  |  |  |  |
| **Cri** |  |  |  |  |  |  |  |  |  |  |
| Serve H, 2013 | Cal | ✓ | Cal | ✓ |  |  |  |  |  |  |
| Perl AE, 2019 | ✓ | ✓ | ✓ | ✓ |  |  |  |  |  |  |
| Cortes JE, 2019 | ✓ | ✓ | ✓ | ✓ |  |  |  |  |  |  |
| **CRp** |  |  |  |  |  |  |  |  |  |  |
| Levis M, 2011 | ✓ | ✓ | ✓ | ✓ |  |  | ✓ |  |  |  |
| Perl AE, 2019 | ✓ | ✓ | ✓ | ✓ |  |  |  |  |  |  |
| Cortes JE, 2019 | ✓ | ✓ | ✓ | ✓ |  |  |  |  |  |  |
| **Overall response rate (CR + CRi)** |  |  |  |  |  |  |  |  |  |  |
| ServeH, 2013 | Cal | ✓ | Cal | ✓ |  |  | ✓ |  |  |  |
| Knapper S, 2017 (AML15) | ✓ | ✓ | ✓ | ✓ | ✓ | ✓ | ✓ | ✓ | ✓ |  |
| Knapper S, 2017 (AML17) | ✓ | ✓ | ✓ | ✓ | ✓ | ✓ | ✓ | ✓ | ✓ |  |
| Cortes JE, 2019 | ✓ | ✓ | ✓ | ✓ |  |  |  |  |  |  |
| **Overall response rate (CR + CRp)** |  |  |  |  |  |  |  |  |  |  |
| Levis M, 2011 | ✓ | ✓ | ✓ | ✓ |  |  | ✓ |  |  |  |
| **30-day mortality** |  |  |  |  |  |  |  |  |  |  |
| Rollig C, 2015 | ✓ | ✓ | ✓ | ✓ |  |  |  |  |  |  |
| Knapper S, 2017 (AML15) | ✓ | ✓ | ✓ | ✓ |  |  |  |  |  |  |
| Knapper S, 2017 (AML17) | ✓ | ✓ | ✓ | ✓ |  |  |  |  |  |  |
| Levis M, 2011 | ✓ | ✓ | ✓ | ✓ |  |  |  |  |  |  |
| Perl AE, 2019 | Cal | ✓ | Cal | ✓ |  |  |  |  |  |  |
| **60-day mortality** |  |  |  |  |  |  |  |  |  |  |
| Rollig C, 2015 | ✓ | ✓ | ✓ | ✓ |  |  |  |  |  |  |
| Serve H, 2013 | ✓ | ✓ | ✓ | ✓ |  |  |  |  |  |  |
| Knapper S, 2017 (AML15) | ✓ | ✓ | ✓ | ✓ |  |  |  |  |  |  |
| Knapper S, 2017 (AML17) | ✓ | ✓ | ✓ | ✓ |  |  |  |  |  |  |
| Stone RM, 2017 | ✓ | ✓ | ✓ | ✓ |  |  |  |  |  |  |
| Perl AE, 2019 | Cal | ✓ | Cal | ✓ |  |  |  |  |  |  |
| **Early death** |  |  |  |  |  |  |  |  |  |  |
| Rollig C, 2015 | ✓ | ✓ | ✓ | ✓ |  |  |  |  |  |  |
| Serve H, 2013 | Cal | ✓ | Cal | ✓ |  |  |  |  |  |  |
| **Treatment-related mortality** |  |  |  |  |  |  |  |  |  |  |
| Rollig C, 2015 | ✓ | ✓ | ✓ | ✓ |  |  |  |  |  |  |

*EFS was estimated for Knapper S (AML15 and AML17).

Abbreviations: cal, calculated; CI, confidence interval; CRi, complete remission with incomplete haematologic recovery; CRp, complete remission with incomplete platelet recovery; FLT3, fms-like tyrosine kinase 3; HR, hazard ratio; N/n, number of; n/a, not applicable; O-E, observed minus expected; pt/s, patient/s.


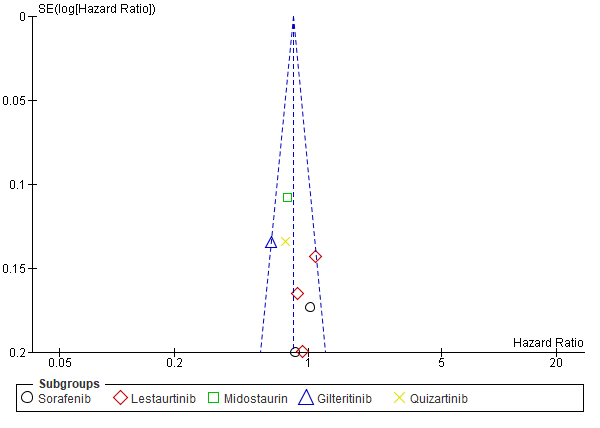


Supplementary Figure 1: Funnel plot of all eight included trials for overall survival


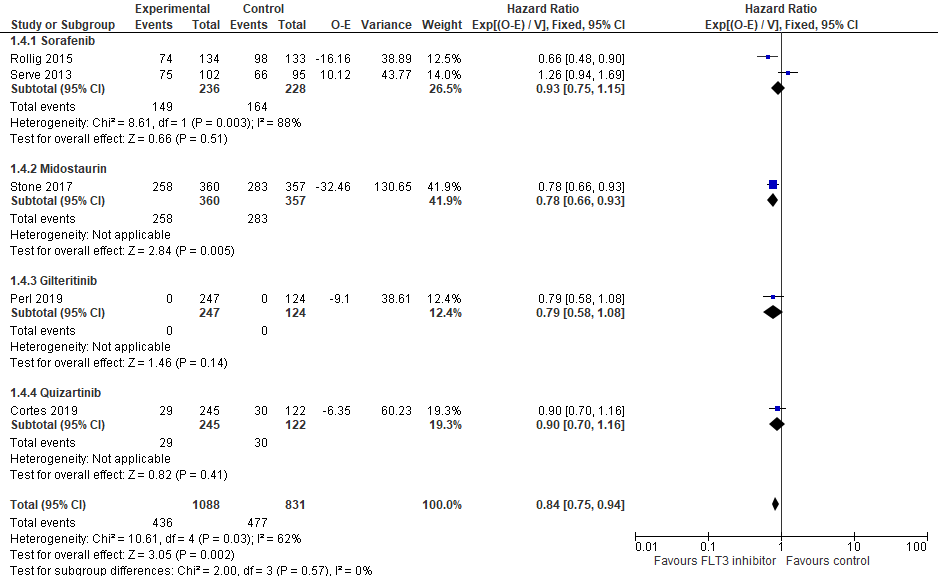


Supplementary Figure 2: Forest plot of event-free survival data (uncensored population), grouped by FLT3 inhibitor


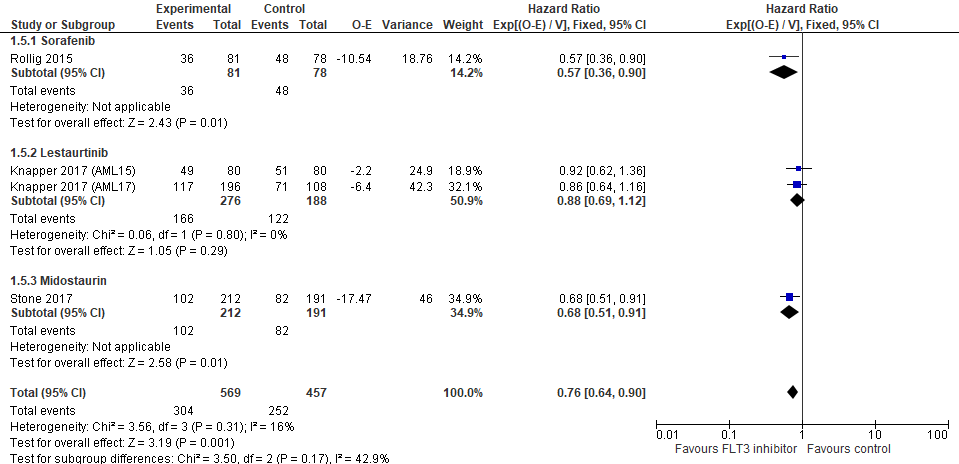


Supplementary Figure 3: Forest plot of relapse-free survival data (uncensored population), grouped by FLT3 inhibitor


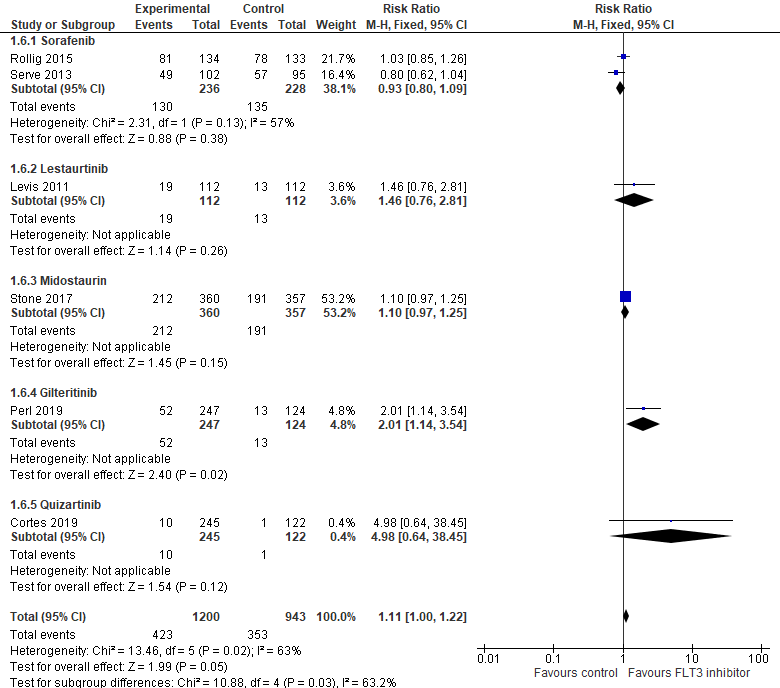


Supplementary Figure 4: Forest plot of complete remission data, grouped by FLT3 inhibitor


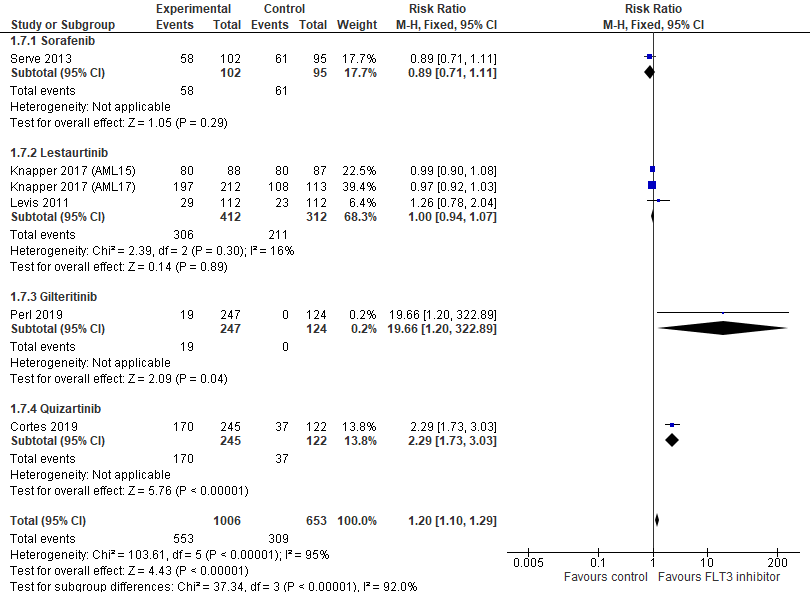


Supplementary Figure 5: Forest plot of overall response rate (CR + CRi/CRp) data, grouped by FLT3 inhibitor


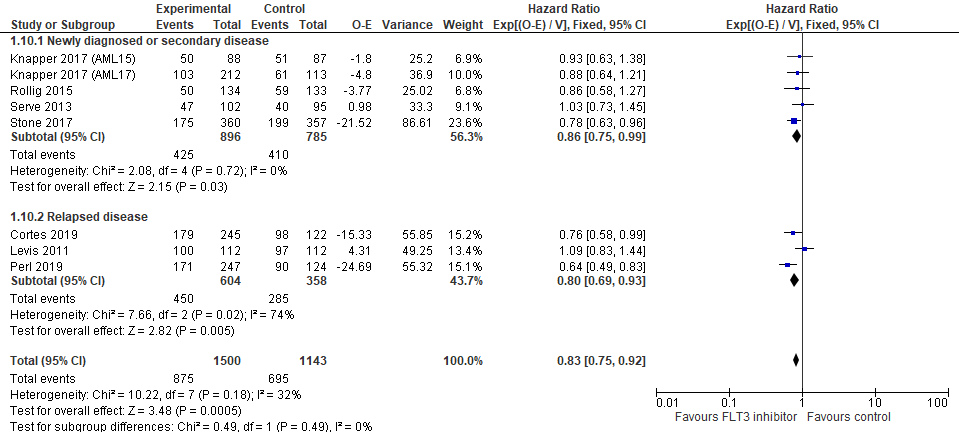


Supplementary Figure 6: Forest plot of overall survival data, grouped by disease stage


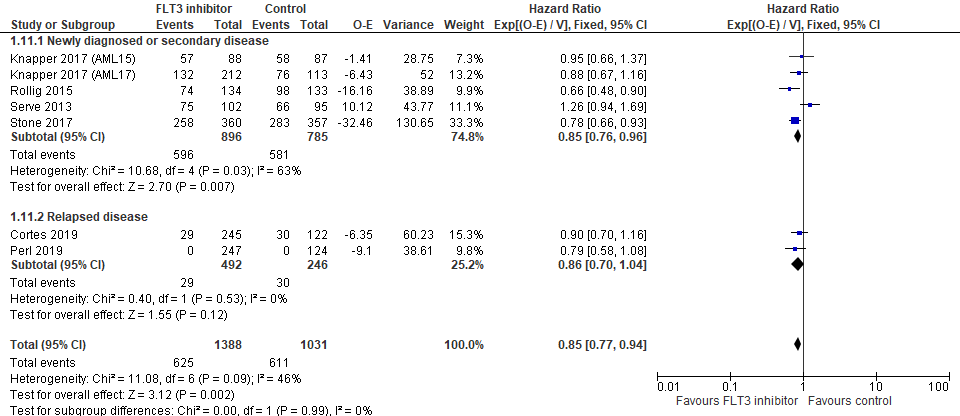


Supplementary Figure 7: Forest plot of event-free survival (calculated and reported data), grouped by disease stage


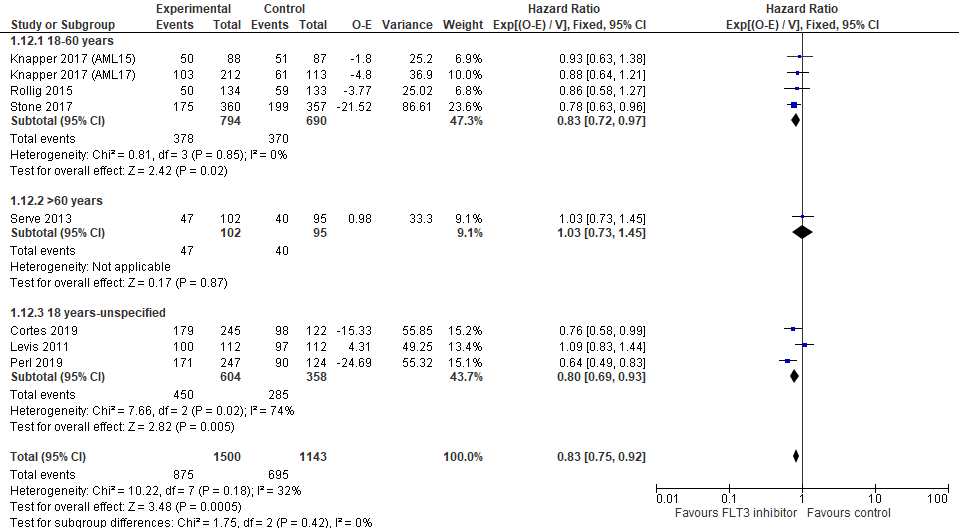


Supplementary Figure 8: Forest plot of overall survival, grouped by age categories


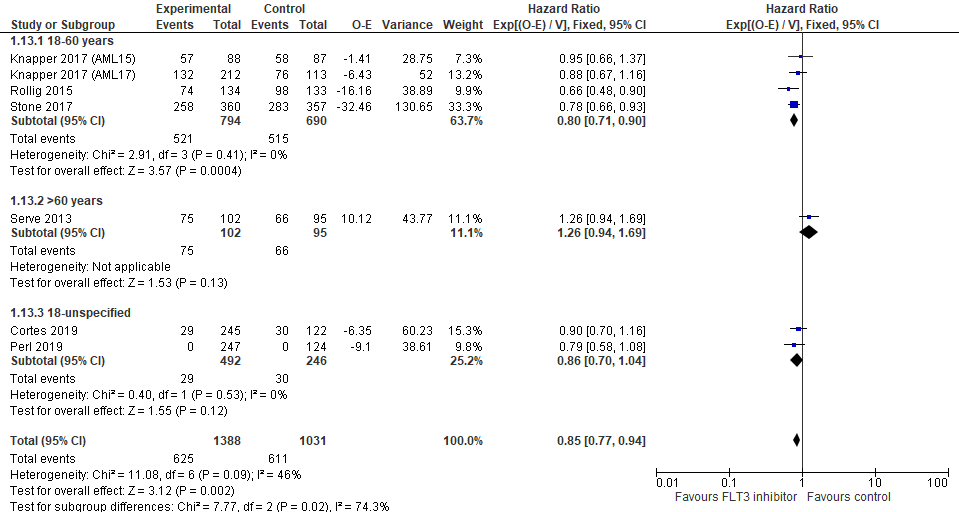


Supplementary Figure 9: Forest plot of event-free survival (calculated and reported data), grouped by age categories

Supplementary Table 7: Baseline and outcome data for FLT3-mutated patients from Rollig C, 2015

| **ROLLIG C, 2015** | | |
| --- | --- | --- |
|  | **SORAFENIB (N=134)** | **PLACEBO (N=133)** |
| **BASELINE n (% or range)** | | |
| NPM1 mutation   - Missing | 43 (32%)  3 (2%) | 43 (32%)  1 (1%) |
| FLT3-ITD mutation   - Missing | 23 (17%)  1 (1%) | 23 (17%)  0 |
| FLT3-ITD/wild type ratio | 0.47 (0.26-0.74) | 0.49 (0.22-0.84) |
|  | | |
| **RESULTS – FLT3-ITD patients only (n=46)** | | |
| Survival (median) | | |
|  | N=23 | N=23 |
| Overall survival | Not reached | 19 mths (95% CI: 0-39) |
| Event-free survival | 5 mths (95% CI: 0-12) | 6 mths (95% CI: 1-11) |
| Relapse-free survival | 18 mths (95% CI: 0-36) | 6 mths (95% CI: 0-16) |

Abbreviations: CI, confidence interval; FLT3-ITD, FLT3-internal tandem duplication; mths, months; NPM1, nucleophosmin 1.

Supplementary Table 8: Baseline and outcome data for FLT3-mutated patients from Serve H, 2013

| **SERVE H, 2013** | | |
| --- | --- | --- |
|  | **SORAFENIB (N=102)** | **PLACEBO (N=95)** |
| **BASELINE** | | |
| FLT3-ITD positive | 15 (15%) | 13 (14%) |
| NPM1 mutated | 21 (21%) | 18 (19%) |
|  |  |  |
| **RESULTS** | | |
| ***Overall survival*** | | |
| FLT3-ITD positive | No significant difference (p=0.21) | |
| FLT3-ITD negative | No significant difference (no further details specified) | |
| NPM1 mutated positive | No significant difference (no further details specified) | |
| NPM1 Mutated negative | No significant difference (no further details specified) | |
|  |  | |
| ***Event-free survival*** | | |
| FLT3-ITD positive | No significant difference (p=0.72) | |
| FLT3-ITD negative | No significant difference (no further details specified) | |
| NPM1 mutated positive | No significant difference (no further details specified) | |
| NPM1 Mutated negative | No significant difference (no further details specified) | |
|  | | |
| ***CR rate*** | | |
| FLT3-ITD positive | 40% | 77% |
|  | No significant difference | |
|  | | |
| ***CR with incomplete recovery rate*** | | |
| FLT3-ITD positive | 20% | 0% |
|  | No significant difference | |
|  | | |
| ***Partial remission rate*** | | |
| FLT3-ITD positive | 0% | 0% |
|  | No significant difference | |
|  | | |
| ***Refractory disease rate*** | | |
| FLT3-ITD positive | 20% | 23% |
|  | No significant difference | |
|  | | |
| ***Early death rate*** | | |
| FLT3-ITD positive | 20% | 0% |
|  | No significant difference | |

Abbreviations: CR, complete remission; FLT3-ITD, FLT3-internal tandem duplication; NPM1, nucleophosmin 1.

Supplementary Table 9: Baseline data for FLT3-mutated patients from Knapper S, 2017

| **KNAPPER S, 2017** | | | | |
| --- | --- | --- | --- | --- |
|  | **AML15** | | **AML17** | |
|  | **LESTAURTINIB (N=88)** | **CONTROL**  **(N=87)** | **LESTAURTINIB**  **(N=212)** | **PLACEBO**  **(N=113)** |
| **BASELINE** | | | | |
| **FLT3 mutation status n (%)** | | | | |
| ITD alone | 65 (74%) | 65 (75%) | 155 (73%) | 85 (75%) |
| TKD alone | 22 (25%) | 18 (21%) | 52 (25%) | 23 (20%) |
| ITD + TKD | 1 (1%) | 2 (2%) | 4 (1.5%) | 4 (4%) |
| Not assessable | 0 | 2 (2%) | 1 (0.5%) | 1 (1%) |
|  |  |  |  |  |
| **ITD mutant n (%)** | | | | |
| - <25% | 18/66 (27%) | 22/67 (33%) | 55/159 (35%) | 31/89 (35%) |
| - 25-50% | 38/66 (58%) | 22/67 (33%) | 77/159 (48%) | 47/89 (53%) |
| - 50%+ | 5/66 (7.5%) | 14/67 (21%) | 27/159 (17%) | 11/89 (12%) |
| - Unknown | 5/66 (7.5%) | 9/67 (13%) | 0 | 0 |
| Median | 32.5 | 36.5 | 29.5 | 31 |
| Range | 5.8 – 92.5 | 3-98.4 | 5 – 98 | 3.5 – 96 |
|  |  |  |  |  |
| **NPM1c status n (%)** | | | | |
| Wild type | 42 (48%) | 34 (39%) | 83 (39%) | 52 (46%) |
| Mutant | 43 (49%) | 45 (52%) | 124 (58%) | 58 (51%) |
| Not known | 3 (3%) | 8 (9%) | 5 (3%) | 3 (4%) |
|  | | | | |

Abbreviations: FLT3, fms-like tyrosine kinase 3; FLT3-ITD, internal tandem duplication; NPM1c, nucleophosmin 1 (cytoplasmic location); TKD, tyrosine kinase domain.

Supplementary Table 10: Outcome data for FLT3-mutated patients from Knapper S, 2017

| **RESULTS (AML15, AML17)** | | | | | |
| --- | --- | --- | --- | --- | --- |
| Stratum | Deaths / Patients | | O-E | Variance | OR (95% CI)  (<1 favours lestaurtinib) |
|  | Lestaurtinib | Control |  |  |  |
| ***Relapse-free survival*** | | | | | |
| ***FLT3-ITD*** | | | | | |
| - Wild type | 39/71 (55%) | 21/41 (51%) | 0.9 | 13.7 | 1.07 (0.63, 1.81) |
| - Mutant | 81/130 (62%) | 58/87 (67%) | -5.3 | 32.1 | 0.85 (0.60, 1.20) |
|  | | | | | |
| ***ITD allele burden*** | | | | | |
| Wild type | 39/71 (55%) | 21/41 (51%) | 0.9 | 13.7 | 1.07 (0.63, 1.81) |
| ITD mutant <25% | 42/67 (63%) | 35/50 (70%) | -4.6 | 17.7 | 0.77 (0.49, 1.23) |
| ITD mutant 25-50% | 60/102 (59%) | 44/66 (67%) | -4.3 | 24.4 | 0.84 (0.57, 1.25) |
| ITD mutant 50%+ | 19/30 (63%) | 19/23 (83%) | -3.1 | 7.4 | 0.66 (0.32, 1.35) |
|  | | | | | |
| ***FLT3-TKD*** | | | | | |
| - Wild type | 80/112 (71%) | 97/139 (70%) | -9.2 | 51.6 | 0.84 (0.64, 1.10) |
| - Mutant | 82/157 (52%) | 57/99 (58%) | -3.3 | 31.8 | 1.0 (0.60, 1.66) |
|  | | | | | |
| ***NPM1c*** | | | | | |
| - Wild type | 80/112 (71%) | 59/79 (75%) | -3.8 | 33.1 | 0.89 (0.63, 1.25) |
| - Mutant | 82/157 (52%) | 57/99 (58%) | -3.3 | 31.8 | - 1. 0.64, 1.28) |
|  | | | | | |
| ***Overall survival*** | | | | | |
| ***FLT3-ITD*** | | | | | |
| - Wild type | 29/74 (39%) | 18/42 (43%) | -1.0 | 10.7 | 0.91 (0.50, 1.66) |
| - Mutant | 123/225 (55%) | 93/156 (60%) | -5.1 | 50.8 | 0.90 (0.69, 1.19) |
|  | | | | | |
| ***ITD allele burden*** | | | | | |
| - Wild type | 29/74 (39%) | 18/42 (43%) | -1.0 | 10.7 | 0.91 (0.50, 1.68) |
| - ITD mutant <25% | 39/73 (53%) | 33/53 (62%) | -4.7 | 16.7 | 0.76 (0.47, 1.22) |
| - ITD mutant 25-50% | 61/115 (53%) | 36/69 (52%) | 0.8 | 22.7 | 1.04 (0.69, 1.56) |
| - ITD mutant 50%+ | 19/32 (59%) | 20/25 (80%) | -2.4 | 8.1 | 0.74 (0.37, 1.47) |
|  | | | | | |
| ***FLT3-TKD*** | | | | | |
| - Wild type | 121/220 (55%) | 90/150 (60%) | -4.8 | 49.6 | 0.91 (0.69, 1.20) |
| - Mutant | 31/79 (39%) | 21/47 (45%) | -1.8 | 11.9 | 0.86 (0.49, 1.52) |
|  | | | | | |
| ***NPM1c*** | | | | | |
| - Wild type | 75/125 (60%) | 57/86 (66%) | -6.8 | 31.0 | 0.80 (0.56, 1.14) |
| - Mutant | 73/167 (44%) | 48/103 (47%) | 0.6 | 28.0 | 1.02 (0.71, 1.48) |

Abbreviations: CI, confidence interval; FLT3-ITD, FLT3-internal tandem duplication; FLT3-TKD, FLT3-tyrosine kinase domain; NPM1c, nucleophosmin 1 (cytoplasmic location); O-E, observed minus expected; OR, odds ratio.

Supplementary Table 11: Baseline and outcome data for FLT3-mutated patients from Levis M, 2011

| **LEVIS M, 2011** | | |
| --- | --- | --- |
|  | **LESTAURTINIB (N=112)** | **CHEMO ONLY (N=112)** |
| **BASELINE N (%)** | | |
| FLT3-ITD only | 101 (90.2%) | 97 (86.6%) |
| D835 only | 9 (8%) | 8 (7.1%) |
| FLT3-ITD and D835 | 2 (1.8%) | 6 (5.4%) |
| Not confirmed | 0 | 1 (0.9%) |
|  | | |
| **RESULTS** |  |  |
| ‘For patients with a D835 mutation only, 5 of 9 achieved a CR/CRp in the lestaurtinib arm, compared with 2 of 8 in the control arm (P=0.6)’. | | |

Abbreviations: CR, complete remission; CRp, complete remission with incomplete platelet recovery; FLT3-ITD, FLT3-internal tandem duplication.

Supplementary Table 12: Baseline and outcome data for FLT3-mutated patients from Stone RM, 2017

| **STONE RM, 2017** | | |
| --- | --- | --- |
|  | **MIDOSTAURIN (N=360)** | **PLACEBO (N=357)** |
| **BASELINE** | | |
| TKD | 81 (22.5%) | 81 (22.7%) |
| ITD with low allelic ratio (0.05 to 0.7) | 171 (47.5%) | 170 (47.6%) |
| ITD with high allelic ratio (>0.7) | 108 (30%) | 106 (29.7%) |
|  | | |
| **RESULTS** | | |
| Survival – HR (95% CI); HR < 1 favours treatment | | |
|  | **OS** | **EFS** |
| Overall | N=717  0.78 (0.63 – 0.96)  1 sided P=0.009 | N=717  0.78 (0.66 – 0.93)  1 sided P=0.002 |
| TKD | N=162  0.65 (0.39 – 1.08)  2 sided P=0.10 | N=162  0.64 (0.43 – 0.96)  2 sided P=0.03 |
| ITD with low allelic ratio (0.05 to 0.7) | N=341  0.81 (0.60 – 1.11)  2 sided P=0.19 | N=341  0.85 (0.67 – 1.09)  2 sided P=0.21 |
| ITD with high allelic ratio (>0.7) | N=214  0.80 (0.57 – 1.12)  2 sided P=0.19 | N=214  0.77 (0.57 – 1.04)  2 sided P=0.08 |

Abbreviations: CI, confidence interval; EFS, event-free survival; HR, hazard ratio; ITD, internal tandem duplication; OS, overall survival; TKD, tyrosine kinase domain.

Supplementary Table 13: Baseline and outcome data for FLT3-mutated patients from Perl AE, 2019

| **PERL AE, 2019** | | |
| --- | --- | --- |
|  | **GILTERITINIB (N=247)** | **SALVAGE CHEMO (N=124)** |
| **BASELINE n (%)** | | |
| FLT3-ITD mutation | 215 (87.0%) | 113 (91.1%) |
| FLT3-TKD mutation | 21 (8.5%) | 10 (8.1%) |
| FLT3-ITD and TKD mutation | 7 (2.8%) | 0 |
|  | | |
| **RESULTS** | | |
| **FLT3-ITD patients only (n=328)** | | |
|  | N=215 | N=113 |
| Overall survival (median months) | 9.3 | 5.6 |
| Overall survival (HR, 95% CI) | HR=0.623 (95% CI: 0.473 to 0.820) | |
| Complete remission n (%) | 44 (20.5) | 11 (9.7) |
| Complete remission (RD, 95% CI) | RD=10.7 (95% CI 2.4 to 19.1) | |
|  | | |
| **FLT3-TKD patients only (n=31)** | | |
|  | N=21 | N=10 |
| Overall survival (median months) | 8.0 | 5.7 |
| Overall survival (HR, 95% CI) | HR=0.693 (95% CI 0.293 to 1.643) | |
| Complete remission n (%) | 4 (19.0) | 2 (20.0) |
| Complete remission (RD, 95% CI) | RD=-1.0 (95% CI -38.3 to 36.4) | |
|  | | |
| **FLT3-ITD and TKD patients (n=7)** | | |
|  | N=7 | N=0 |
| Overall survival (median months) | 10.2 | Not evaluable |
| Overall survival (HR, 95% CI) | Not evaluable | |
| Complete remission n (%) | 2 (28.6) | Not evaluable |
| Complete remission (RD, 95% CI) | Not evaluable | |
|  | | |
| **Unconfirmed FLT3 mutations (n=5)** | | |
|  | N=4 | N=1 |

Abbreviations: CI, confidence interval; FLT3-ITD, FLT3-internal tandem duplication; FLT3-TKD, FLT3-tyrosine kinase domain; HR, hazard ratio; RD, risk difference.

Supplementary Table 14: Baseline data from FLT3-ITD-mutated patients from Cortes JE, 2019

| **CORTES JE, 2019** | | |
| --- | --- | --- |
| **BASELINE** | | |
| **FLT3-ITD variant allele frequency n (%)** | | |
|  | **QUIZARTINIB (N=245)** | **SALVAGE CHEMO (N=122)** |
| <3% | 3 (1) | 0 |
| ≥3% to ≤25% | 66 (27) | 37 (30) |
| >25% to ≤50% | 86 (35) | 42 (34) |
| >50% | 90 (37) | 43 (35) |

Abbreviations: FLT3-ITD, FLT3-internal tandem duplication.

Supplementary Table 15: Outcome data for FLT3-mutated AML patients from Cortes JE, 2019

| **CORTES JE, 2019** | | | | | |
| --- | --- | --- | --- | --- | --- |
| **RESULTS – Overall survival** | | | | | |
| FLT3-ITD variant allele frequency | QUIZARTINIB | | SALVAGE CHEMO | | HR (95% CI)  (<1 favours quizartinib) |
|  | n/N | Median months (95% CI) | n/N | Median months (95% CI) |  |
| - ≥3% to ≤25% | 47/66 | 7.2 (6.0 to 11.4) | 24/37 | 6.1 (4.6 to 8.8) | 0.88 (0.54 to 1.44) |
| - >25% to ≤50% | 67/86 | 5.7 (4.5 to 7.1) | 32/42 | 4.7 (2.1 to 7.6) | 0.69 (0.45 to 1.05) |
| - >50% | 74/90 | 5.4 (4.1 to 7.3) | 30/43 | 3.5 (1.9 to 4.9) | 0.69 (0.45 to 1.05) |

Abbreviations: CI, confidence interval; FLT3-ITD, FLT3-internal tandem duplication, HR, hazard ratio.


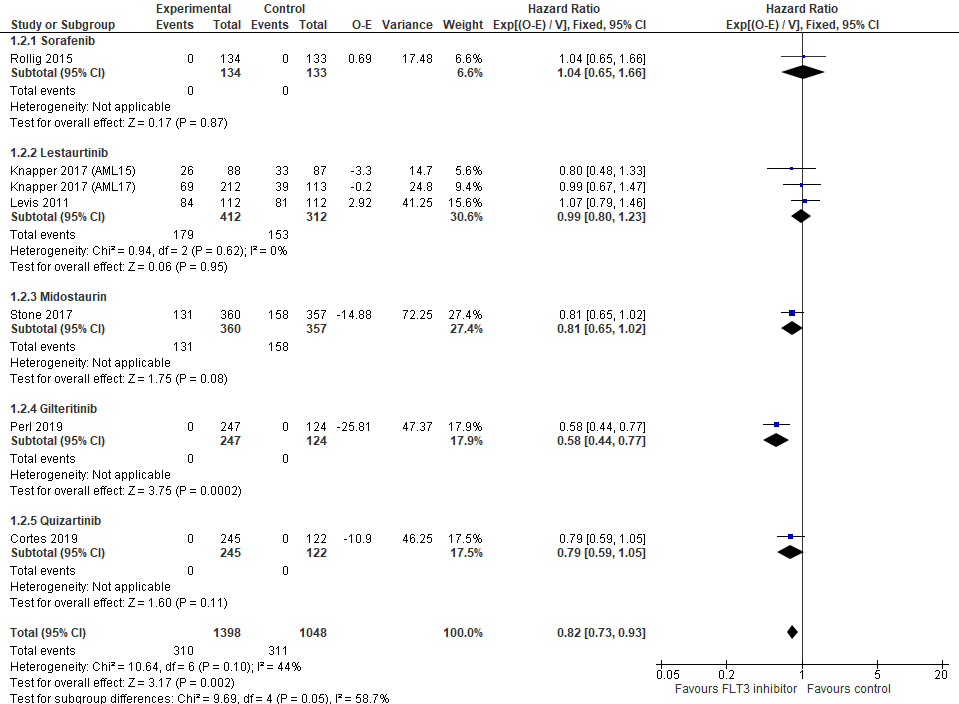


Supplementary Figure 10: Forest plot of overall survival data (censored population), grouped by FLT3 inhibitor


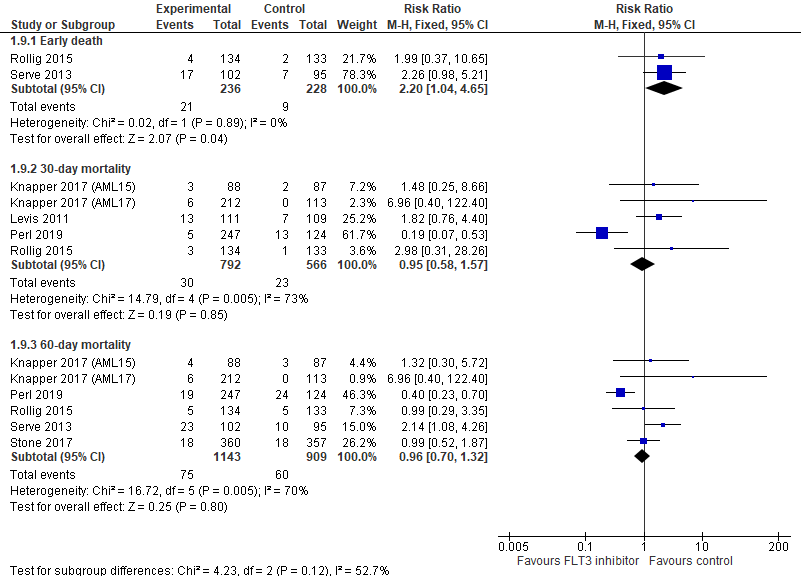


Supplementary Figure 11: Forest plot of early death/mortality data

‘Early death’ was not clearly defined/distinguishable from 30-day mortality in the Rollig trial.


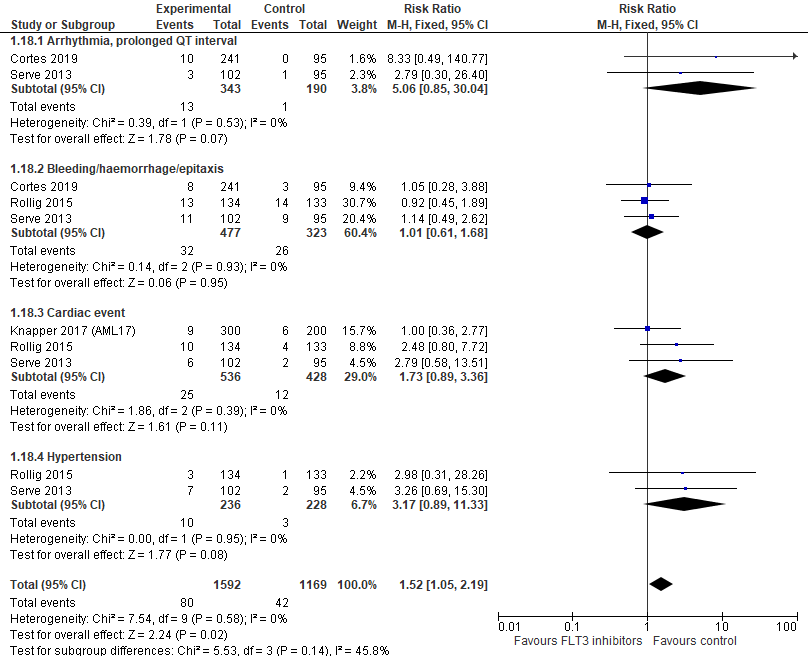


Supplementary Figure 12: Forest plot of vascular adverse events data

Knapper 2017 (AML17) – represents data from both AML15 and AML17 collectively.


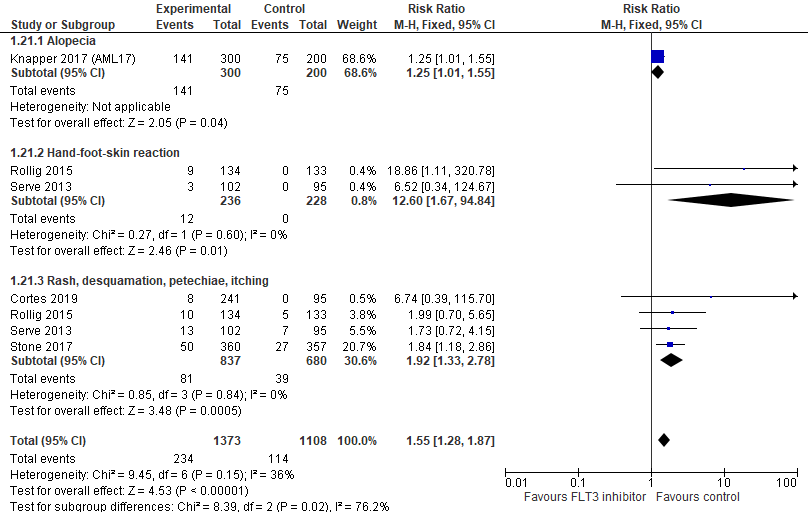


Supplementary Figure 13: Forest plot of dermatological adverse events data

Knapper 2017 (AML17) – represents data from both AML15 and AML17 collectively.


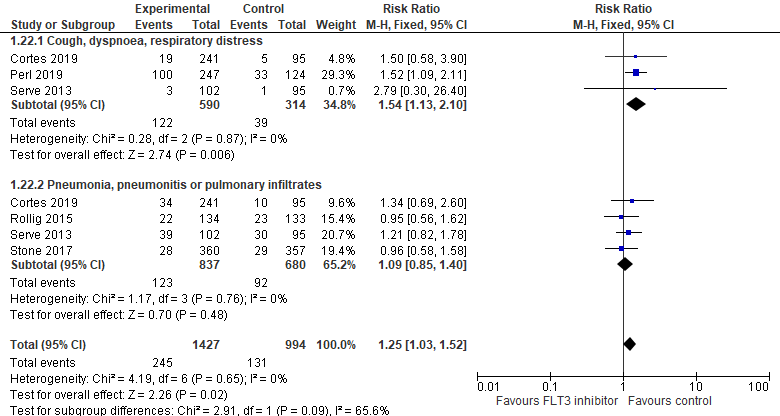


Supplementary Figure 14: Forest plot of respiratory adverse events data


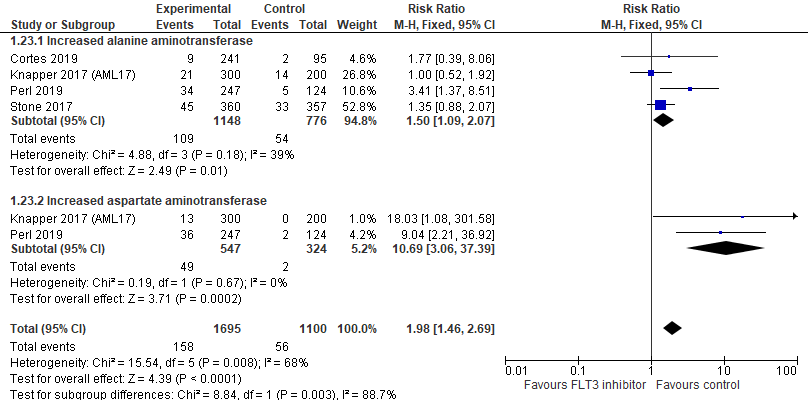


Supplementary Figure 15: Forest plot of liver/hepatobiliary adverse events data

Knapper 2017 (AML17) – represents data from both AML15 and AML17 collectively.


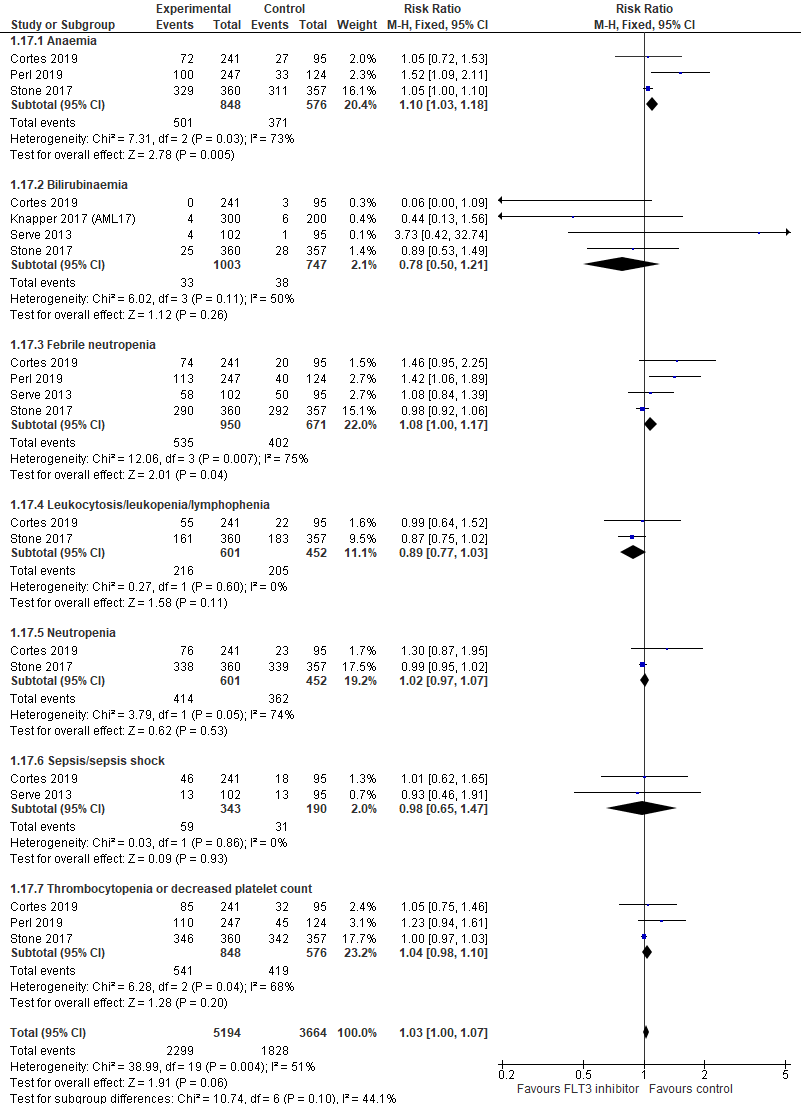


Supplementary Figure 16: Forest plot of haematological adverse events data

Knapper 2017 (AML17) – represents data from both AML15 and AML17 collectively.


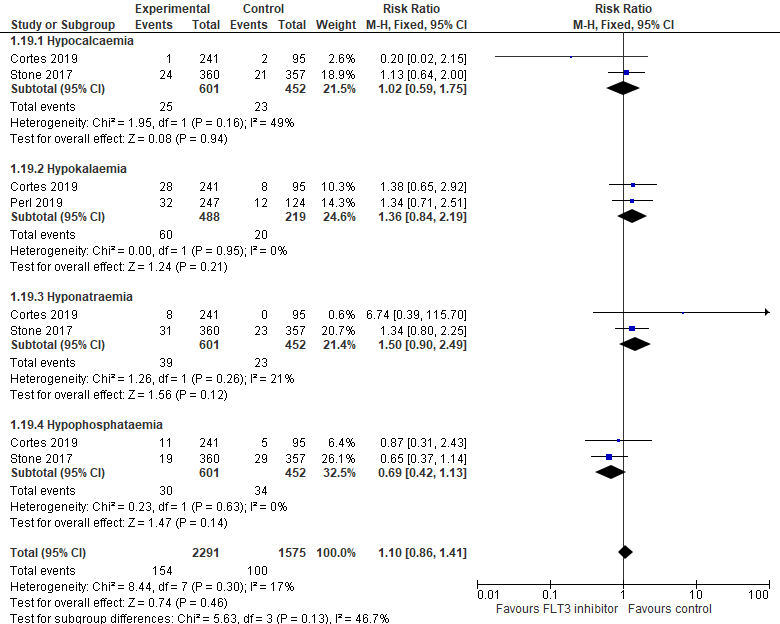


Supplementary Figure 17: Forest plot of metabolism and nutritional disorders data


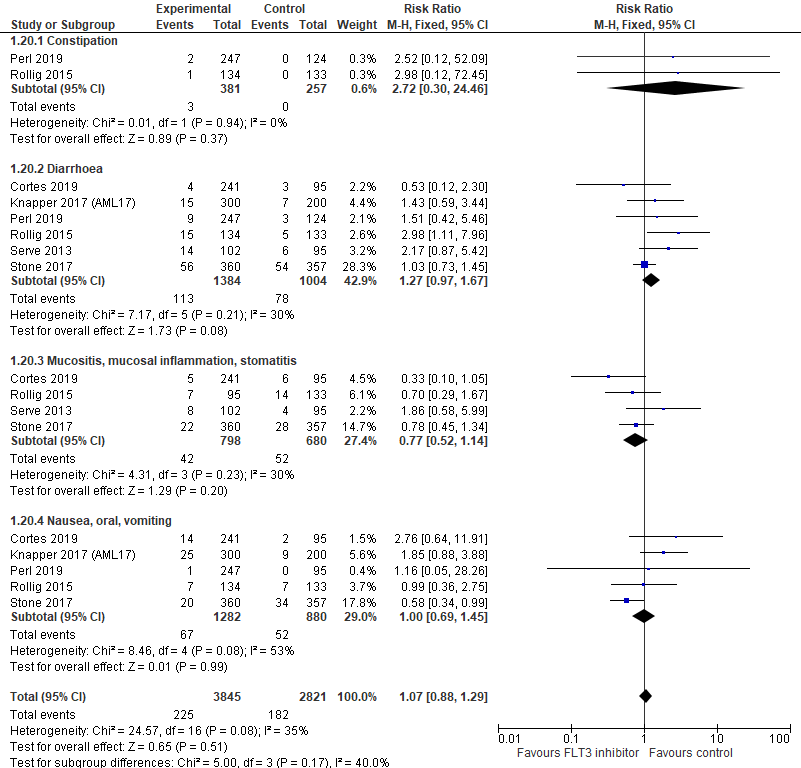


Supplementary Figure 18: Forest plot of gastrointestinal disorders data

Knapper 2017 (AML17) – represents data from both AML15 and AML17 collectively.


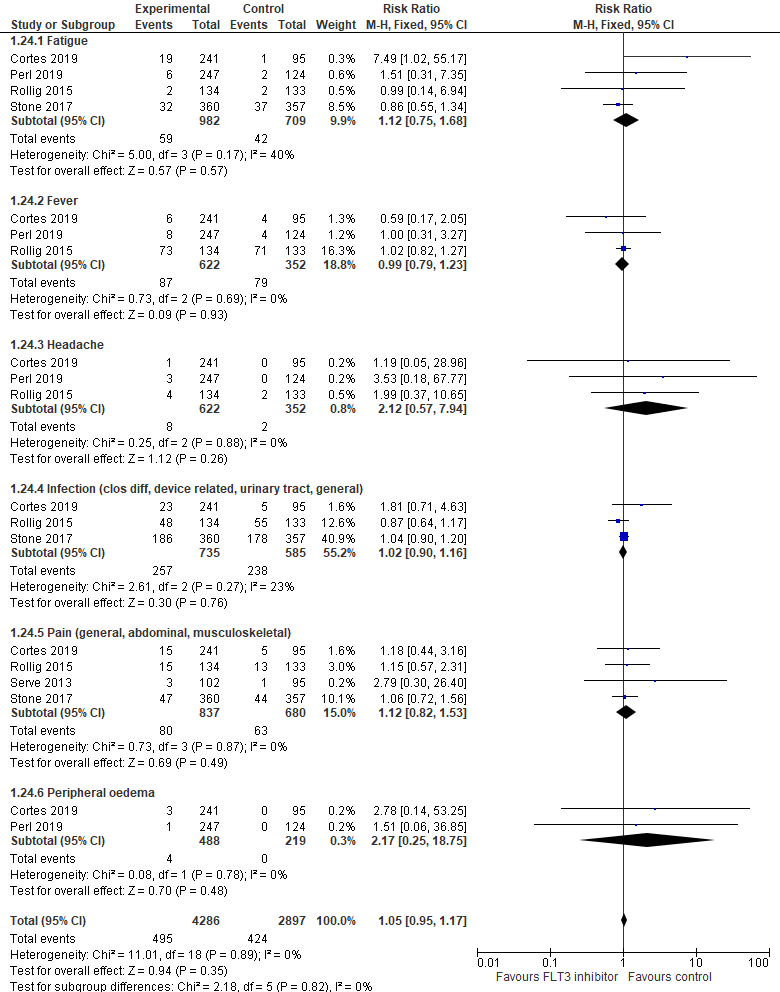


Supplementary Figure 19: Forest plot of constitutional symptoms data


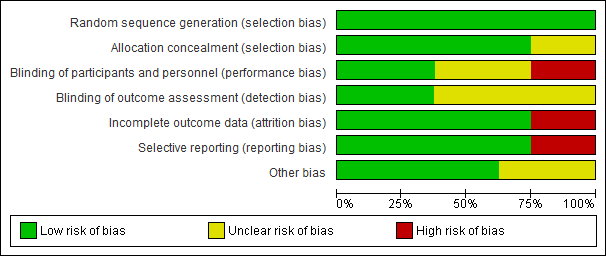


Supplementary Figure 20: Risk of bias graph of included trials


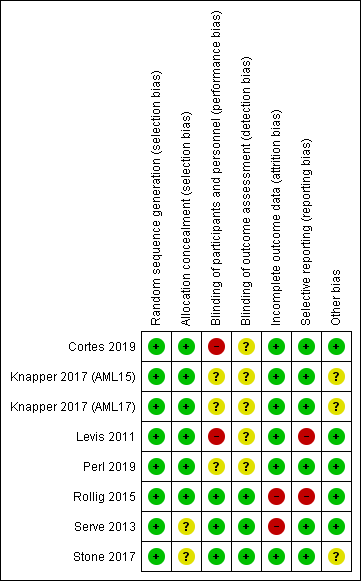


Supplementary Figure 21: Risk of bias summary of included trials
